# Mechanism of Multiple Emission in Gold Quantum Boxes and Design Rules for Strong Near-Infrared Luminescence

**DOI:** 10.1021/acsnano.6c13094

**Published:** 2026-09-22

**Authors:** Kang Li, Yitong Wang, Pu Wang, Rongchao Jin, Yong Pei

**Affiliations:** † Department of Chemistry, Key Laboratory of Environmentally Friendly Chemistry and Applications of Ministry of Education, 12665 Xiangtan University, Xiangtan, Hunan 411105, China; ‡ College of Chemistry & Chemical Engineering, Hunan Normal University, Changsha, Hunan 410081, China; § Department of Chemistry, 311285 Carnegie Mellon University, Pittsburgh, Pennsylvania 15213, United States

**Keywords:** face-centered cubic (FCC) gold quantum boxes, photoluminescence mechanism, nonradiative decay, low-frequency motions, large reorganization energies

## Abstract

Ligand-protected gold nanoclusters (Au NCs) hold great promise for near-infrared photonics and bioimaging, but their low photoluminescence quantum yields (PLQYs) and unclear emission mechanisms have limited the rational design of highly luminescent NCs. Here, we report a comprehensive theoretical investigation of the representative Au_8n+4_(SR)_4n+8_ quantum-box series (n = 3, 4, 5, 6) using density functional theory (DFT) and time-dependent DFT (TD-DFT) calculations to elucidate the structure–photophysics relationship. We reveal that high structural symmetry and small aspect ratios (AR) severely limit transition dipole moments and radiative decay rates in these face-centered-cubic (FCC)-type NCs, in contrast to highly emissive hexagonal close-packed (HCP) quantum-rod analogues. On the other hand, the quantum-box NCs exhibit unusually small singlet–triplet energy gaps (Δ*E*_ST_) and strong spin–orbit coupling, enabling ultrafast intersystem crossing (ISC) and reverse intersystem crossing (RISC), giving rise to simultaneous prompt fluorescence (PF), thermally activated delayed fluorescence (TADF), and phosphorescence. The small gaps arise from spatially decoupled electron–hole distributions distinct from those in organic TADF materials. Moreover, low-frequency ligand (the -R group) torsional and librational motions dominate internal conversion via large reorganization energies, while one-dimensional cluster growth modulates staple-motif packing and induces nonmonotonic, ligand-dominated nonradiative relaxation. Collectively, weak radiative transitions due to symmetry constraints and efficient nonradiative decay via unconstrained ligand vibrations account for the universally low PLQY of quantum-box Au NCs. This work clarifies the fundamental roles of symmetry, anisotropy, and ligand dynamics in luminescence, offering critical design rules for developing high-efficiency near-infrared-emissive Au NCs.

## Graphical abstract

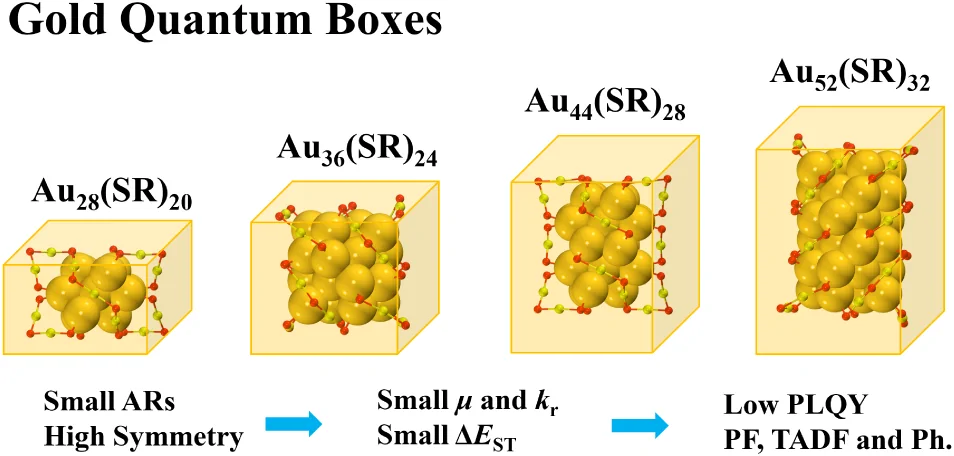

## Introduction

Ligand-protected gold nanoclusters (Au NCs) have emerged as a captivating class of luminescent nanomaterials, owing to their distinctive photoluminescence (PL) properties. Featuring exceptional biocompatibility, negligible cytotoxicity, and long-lived near-infrared (NIR) luminescence, these Au NCs enable deep-tissue optical imaging, thereby positioning them as promising platforms for biomedical imaging, disease diagnostics, and therapeutic applications.^1–3^ Furthermore, NIR-luminescent Au NCs have found applications in optics, such as NIR photon upconversion into visible photons by utilizing long-lived triplet states.^2,3^ Despite substantial advances in the atomically precise synthesis and structural elucidation of Au NCs, their photoluminescence quantum yields (PLQYs) remain generally low, and the fundamental mechanisms governing their emission behavior remain incompletely understood, particularly regarding nonradiative decay mechanisms. This critical knowledge gap represents a major bottleneck for the rational design of highly luminescent Au NCs, especially those emitting in the NIR region, where high-efficiency luminescence is inherently challenging to realize due to significantly accelerated nonradiative decay. Consequently, a systematic interrogation of the intrinsic PL mechanisms is urgently required from both fundamental and applied perspectives.

To enhance the PLQY of ligand-protected Au NCs, diverse synthetic strategies have been explored, including size engineering, heterometal doping,^4,5^ ligand shell modification,^6–8^ and aggregation-induced emission (AIE).^9,10^ While certain approaches successfully widen the HOMO–LUMO energy gap to promote radiative transitions and suppress nonradiative decay channels, such modifications inevitably induce a blue shift in the emission maximum, which compromises their utility for NIR and second near-infrared (NIR-II) bioimaging and photonics. In accordance with the energy gap law,^11^ nonradiative relaxation processes become increasingly dominant as the emission wavelength red-shifts. Thus, the central challenge in developing high-PLQY NIR-emissive Au NCs resides in concurrently suppressing electron–vibration coupling across a narrow energy gap while preserving a large transition dipole moment to sustain a high radiative decay rate.

Recent studies have demonstrated that the optical performance of Au NCs is predominantly governed by the synergistic interplay between the cluster size and structural architecture.^12,13^ Increasing the size of Au NCs effectively narrows the HOMO–LUMO gap, allowing for tunable emission extending into the NIR window.^14–18^ Notably, one-dimensional (1D) ultrathin rod-like Au NCs (e.g., the Au_42_(PET)_32_ to Au_114_(PET)_80_ family, PET = 2-phenylethanethiolate) exhibit a pronounced red shift in absorption with increasing size (i.e., rod’s length),^19^ accompanied by bright sub-nanosecond NIR luminescence spanning 1000–2200 nm.^20,21^ Benefiting from their large aspect ratios (ARs), these anisotropic NCs possess intense optical absorption and large transition dipole moments,^22^ with representative species (e.g., Au_60_(PET)_44_) achieving remarkable PLQYs of up to 20.9% in the NIR-II region.^21^ Intriguingly, these rod-like Au NCs adopt a hexagonal close-packed (HCP) core structure, whereas most previously reported face-centered cubic (FCC)-type Au NCs feature much smaller ARs and drastically lower PLQYs.

Parallel to the HCP rod-like family, the FCC-based Au_8n+4_(TBBT)_4n+8_ series (comprising Au_28_(TBBT)_20_, Au_36_(TBBT)_24_, Au_44_(TBBT)_28_, and Au_52_(TBBT)_32_; TBBT = 4-tert-butylbenzenethiolate) constitutes another prototypical 1D-extended nanostructure family. Although their 1D structural elongation implies potential for efficient NIR luminescence, these clusters, featuring smaller ARs and greater quasi-symmetry, behave like “quantum boxes” distinctly different from the “quantum rods” observed in the HCP series. Despite their structural extension along the 1D direction, the PLQYs of these quantum-box clusters remain exceptionally low (generally below 2%).^23^ To date, a systematic understanding of the structure–PL efficiency relationship underlying this inferior luminescence performance remains elusive. Notably, this unknown mechanism is further compounded by the diverse emission pathways (fluorescence versus phosphorescence) exhibited by these clusters. For instance, the Au_42_(PET)_32_ quantum rod displays fluorescence–phosphorescence dual emission,^24^ while Au_28_(TBBT)_20_ and Au_52_(TBBT)_32_ quantum boxes manifest thermally activated delayed fluorescence (TADF)–phosphorescence dual emission.^25,26^ Although multiple mechanisms have been proposed to rationalize dual/multiple emission in ligand-protected Au NCs,^25,27–32^ the intrinsic correlation between cluster structure and emission type remains unclear. Therefore, a comprehensive investigation of the Au_8n+4_(TBBT)_4n+8_ series is necessary to uncover the fundamental origins of PL quenching in FCC-type Au NCs and clarify the influence of aspect ratio on excited-state properties, ultimately establishing a rational design principle for high-performance NIR-emissive Au NCs.

In this work, we employ density functional theory (DFT) and time-dependent DFT (TD-DFT) calculations to unravel the PL mechanisms of the Au_8n+4_(SR)_4n+8_ quantum-box series, specifically Au_28_(SR)_20_, Au_36_(SR)_24_, Au_44_(SR)_28_, and Au_52_(SR)_32_ (SR is simplified as SCH_3_). Through systematic quantification of radiative decay rates and nonradiative relaxation pathways, we elucidate the intrinsic origins of the universally low PLQY characteristic of these FCC clusters. Our results reveal that the high quasi-symmetry (*D*_2_ and *D*_2d_) and moderate ARs of these quantum boxes give rise to inherently small transition dipole moments, which severely restrict radiative transitions. Concurrently, low-frequency vibrational modes dominated by the alkyl R-groups of peripheral thiolate ligands strongly promote excited-state nonradiative relaxation, further depressing PLQY. Furthermore, we identify a universal multi-emission mechanism involving the coexistence of prompt fluorescence (PF), TADF, and phosphorescence, originating from the exceptionally small singlet–triplet energy gaps (Δ*E*_ST_) in these clusters. Collectively, these findings establish a definitive theoretical correlation between structural characteristics, excited-state dynamics, and PL efficiency in quantum-box Au NCs, providing a strategic framework for the rational design of highly efficient NIR-emissive Au NCs.

## Results and Discussion

### Symmetry-Controlled Radiative Transitions in Au_8n+4_(SCH_3_)_4n+8_ Quantum Boxes

The Au-S skeleton structures of the FCC-based Au_8n+4_(TBBT)_4n+8_ series (n = 3, 4, 5, 6) determined by X-ray crystallography are shown in Figure 1, including Au_28_(TBBT)_20_, Au_36_(TBBT)_24_, Au_44_(TBBT)_28_, and Au_52_(TBBT)_32_. These NCs grow by the sequential addition of one atomic layer and constitute a prototypical 1D-extended nanostructure family.

**Figure 1:**
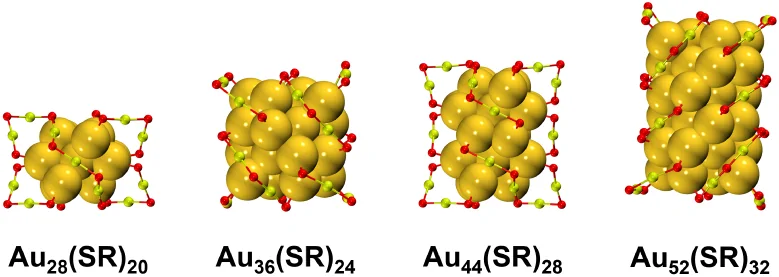
The atomic structures of Au_28_(SR)_20_, Au_36_(SR)_24_, Au_44_(SR)_28_, and Au_52_(SR)_32_. Color code: red balls denote sulfur atoms; large and small yellow balls denote gold atoms in the core and in the staple motifs; R groups are omitted for clarity.

Previous investigations have documented the coexistence of TADF and phosphorescence in Au_28_(TBBT)_20_ and Au_52_(TBBT)_32_,^25,26^ a behavior consistent with the characteristic broad emission bands observed in their steady-state PL spectra.^23,33^ Such spectrally broad emission features imply substantial overlap between fluorescence and phosphorescence channels across the entire Au_8n+4_(TBBT)_4n+8_ series, which severely complicates the experimental deconvolution of individual emissive pathways. Motivated by these experimental observations, we performed full structural optimizations of the lowest singlet-excited (S_1_) and triplet-excited (T_1_) states for the Au_8n+4_(SCH_3_)_4n+8_ NCs, initiated from the fully relaxed ground-state (S_0_) geometries. Vertical emission energies and radiative decay rates were subsequently computed to characterize the excited-state properties.

As summarized in Table 1, the calculated fluorescence emission energies (S_1_ → S_0_) for Au_28_(SCH_3_)_20_, Au_36_(SCH_3_)_24_, and Au_52_(SCH_3_)_32_ show good agreement with the corresponding experimental values, validating the accuracy and reliability of our computational methodology. In contrast, the computed fluorescence emission energy of Au_44_(SCH_3_)_28_ is lower than that of Au_52_(SCH_3_)_32_, deviating from the experimentally observed size-dependent emission trend. Strikingly, Au_44_(TBBT)_28_ displays an unusually broad emission bandwidth, implying the potential contribution of multiple emissive excited-state conformers on its S_1_ potential energy surface (PES). To test this hypothesis, we located an alternative S_1_ local minimum by reoptimizing the S_1_ state using the preoptimized S_2_ geometry as the starting point. This procedure yielded a significantly blue-shifted fluorescence emission peak at 735 nm.

**Table 1:** Computed Emission Properties for the S_1_ → S_0_ and T_1_ → S_0_ Transitions and Experimental Emission Wavelengths of the Quantum Box Clusters^a^

	Au_28_(SCH_3_)_20_	Au_36_(SCH_3_)_24_	Au_44_(SCH_3_)_28_	Au_52_(SCH_3_)_32_
*E*_em_(S_1_→S_0_) (eV)	1.570	1.531	1.355	1.411
*E*_em_(T_1_→S_0_) (eV)	1.349	1.307	1.287	1.408
*λ*_em_(S_1_→S_0_) (nm)	789	809	915	879
*λ*_em_(T_1_→S_0_) (nm)	920	949	964	881
*λ*_em, exp_ (nm)	860	825	900	936
*k*_r_(S_1_ → S_0_) (s^–1^)	3.5 × 10^5^	2.3 × 10^6^	4.5 × 10^5^	1.1 × 10^6^
*k*_r_(T_1_ → S_0_) (s^–1^)	5.8 × 10^3^	1.4 × 10^4^	2.2 × 10^4^	1.5 × 10^4^
*f*_*em*_(S_1_ → S_0_)	0.003	0.022	0.006	0.013

a E_em_(S_1_ → S_0_) and E_em_(T_1_ → S_0_) denote the vertical emission energies calculated at the optimized S_1_ and T_1_ minimum-energy structures, respectively. λ_em_(S_1_ → S_0_) and λ_em_(T_1_ → S_0_) are the corresponding calculated emission wavelengths. λ_em,exp_ denotes the experimentally measured emission wavelength. *k*_r_(S_1_ → S_0_) and *k*_r_(T_1_ → S_0_) are the calculated radiative rate constants for the singlet and triplet emission processes, respectively, and *f*_em_(S_1_ → S_0_) is the oscillator strength of the S_1_ → S_0_ emission transition.

Further structural analysis reveals that the two distinct S_1_ local minima derived from S_0_ and S_2_ initial geometries (Figure S1, panel B) differ by 0.33 eV in vertical emission energy but by only 0.09 eV in adiabatic energy, with a small atomic root-mean-square deviation (RMSD) of 0.18 Å. These findings confirm that the S_1_ PES of Au_44_(SCH_3_)_28_ accommodates multiple closely spaced local minima, and the collective emission from these discrete minima accounts for its experimentally observed broad emission band. By contrast, identical re-optimization protocols applied to the larger Au_52_(SCH_3_)_32_ cluster did not uncover any additional S_1_ local minima, indicating a more rigid, single-well S_1_ PES for this nanocluster. Finally, although the computed phosphorescence peaks are systematically red-shifted relative to fluorescence peaks by up to ∼140 nm, the intrinsically broad emission bands of these nanoclusters lead to substantial spectral overlap, rendering the experimental discrimination of fluorescence and phosphorescence bands exceedingly challenging at room temperature. Nevertheless, the low-temperature PL spectra of Au_28_(SCH_3_)_20_, Au_36_(SCH_3_)_24_, and Au_44_(SCH_3_)_28_ (Figure S2) further split into two (or even three) emission peaks, suggesting the coexistence of multiple emissive channels.

With respect to radiative efficiency, both fluorescence and phosphorescence radiative rate constants for this series of NCs are uniformly low, consistent with their experimentally measured low PLQYs. Notably, among the investigated Au_8n+4_(SCH_3_)_4n+8_ quantum-box NCs, Au_36_(SCH_3_)_24_ exhibits the largest oscillator strength (0.022) for the S_1_ → S_0_ transition. Its emission energy is second only to that of Au_28_(SCH_3_)_20_, affording the highest radiative rate constant within the series. Given that oscillator strength scales with the square of the transition dipole moment, we further mapped the real-space spatial distributions of the transition dipole moment density (TDMD) along the x, y, and z Cartesian axes for the S_1_ → S_0_ transition in the Au_8n+4_(SCH_3_)_4n+8_ quantum-box NCs (Figure 2 and Figure S3). For a direct comparison, the calculated TDMD of the HCP-structured quantum-rod analog Au_42_(SCH_3_)_32_ was also included as a benchmark.

**Figure 2:**
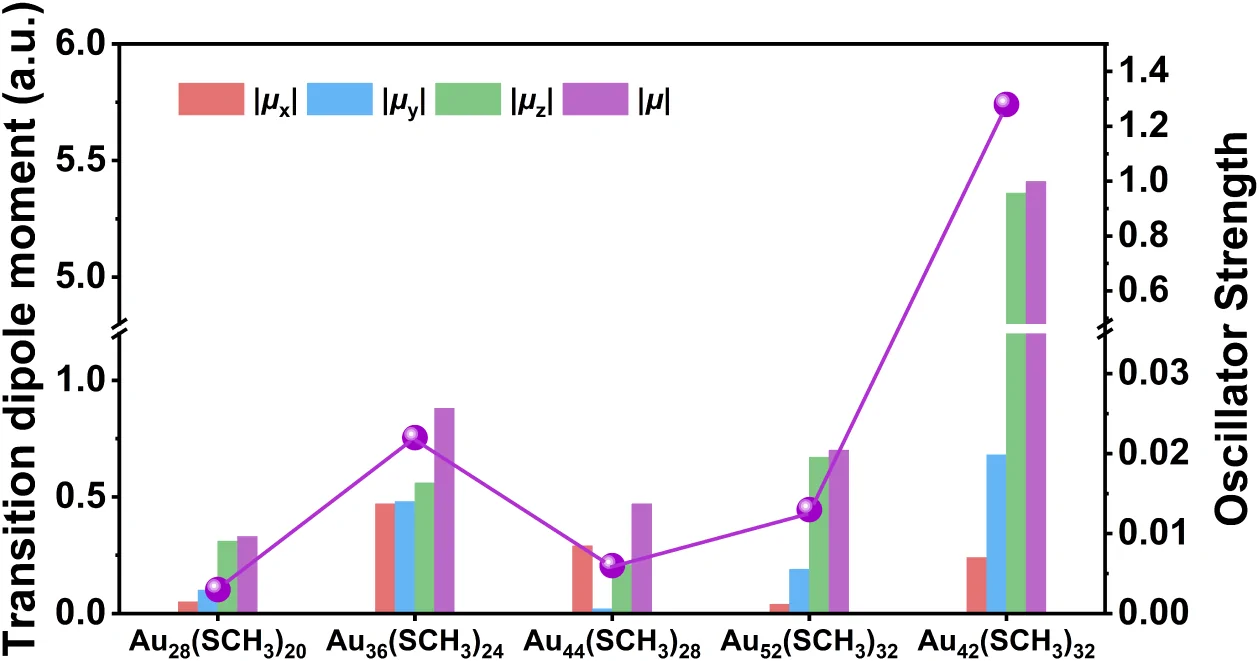
Calculated Cartesian components, absolute values of the transition dipole moment (|*μ*_x_|, |*μ*_y_|, and |*μ*_z_|), the total |*μ*| and the oscillator strength for Au_28_(SCH_3_)_20_, Au_36_(SCH_3_)_24_, Au_44_(SCH_3_)_28_, Au_52_(SCH_3_)_32_, and Au_42_(SCH_3_)_32_.

Although the S_1_ → S_0_ transitions of the Au_8n+4_(SCH_3_)_4n+8_ quantum-box NCs are formally symmetry-allowed, their relatively high point-group symmetries (quasi-*D*_*2*_ or *D*_*2d*_) induce TDMD distributions with alternating positive and negative phases in real space. Because these positive and negative contributions exhibit comparable magnitudes but opposite signs, extensive cancellation occurs upon spatial integration. As a direct consequence, the net transition dipole moment is severely diminished, leading to intrinsically low oscillator strengths and thus weak radiative transitions.

In sharp contrast, the quantum-rod cluster Au_42_(SCH_3_)_32_ displays an exceptionally large oscillator strength (*f* = 1.28). Along its longitudinal (z) axis, the TDMD exhibits a unidirectional sign with a spatially continuous distribution, which eliminates positive–negative cancellation during integration and accordingly yields a large net transition dipole moment. This behavior is strongly correlated with the much lower point-group symmetry (*C*_*i*_, with its character table provided in Table S3) of Au_42_(SCH_3_)_32_. Further comparative analysis reveals that while the Au_8n+4_(SCH_3_)_4n+8_ clusters adopt overall one-dimensionally extended geometries, with their dominant transition dipole moment components aligned preferentially along the elongation (z) axis (Figures 2 and 3), their relatively high structural symmetries impose strict phase constraints on the electronic transition dipole moment. These symmetry-imposed restrictions impede the in-phase accumulation of the TDMD along the z direction. As a result, the total transition dipole moment and its axial component do not increase monotonically with cluster-size extension; instead, they exhibit pronounced oscillations with increasing size. Moreover, the relatively modest ARs of the Au_8n+4_(SCH_3_)_4n+8_ clusters further restrict the magnitude of the z component and the overall transition dipole moment.

**Figure 3:**
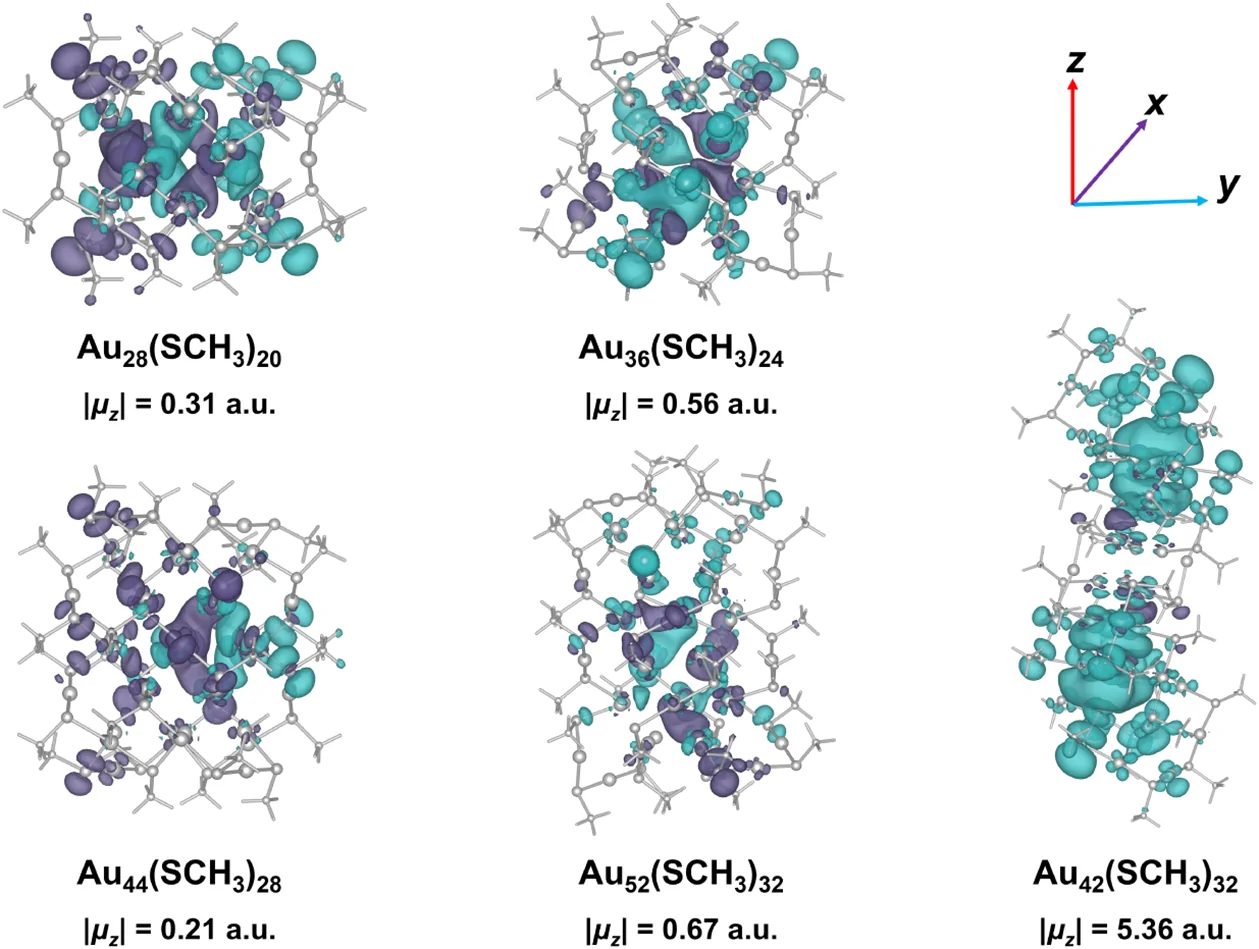
Isosurface representations of the TDMD along *the z* direction for Au_8n+4_(SCH_3_)_4n+8_ and Au_42_(SCH_3_)_32_ clusters. Cyan and purple isosurfaces denote the positive and negative regions of the TDMD. All isosurfaces are plotted at an identical isovalue of ±0.001 a.u. The cluster framework is shown as light gray sticks for clarity.

Of particular note, the nearly cubic Au_36_(SCH_3_)_24_ cluster possesses comparably large transition dipole moment components along all three Cartesian directions, a direct consequence of its quasi-*D*_*2d*_ symmetry. For Au_28_(SCH_3_)_20_, Au_44_(SCH_3_)_28_, and Au_52_(SCH_3_)_32_, which belong to the *D*_2_ point group, the x-, y-, and z-components of the TDMD transform as distinct irreducible representations (Table S1). This symmetry assignment indicates that the magnitudes of these components are symmetry-independent and not constrained to be equal. By contrast, within the *D*_2d_ point group of Au_36_(SCH_3_)_24_, the x- and y-TDMD components belong to the same two-dimensional irreducible representation E and are therefore symmetry-equivalent (Table S2), yielding nearly identical magnitudes along x and y (0.47 and 0.48 a.u., respectively). The simultaneous presence of three substantial transition dipole moment components ultimately gives rise to the largest total transition dipole moment for Au_36_(SCH_3_)_24_ among the Au_8n+4_(SCH_3_)_4n+8_ quantum box clusters.

Collectively, these results establish that the radiative transition rates of ligand-protected Au NCs are governed not merely by geometric ARs but, more fundamentally, by their point-group symmetry. While structural anisotropy dictates the preferential orientation of the transition dipole moment, high symmetry restricts the in-phase buildup of the TDMD, suppresses radiative decay channels, and leads to intrinsically low PLQYs. In essence, symmetry-imposed electronic constraints represent the fundamental origin of radiative rate limitation in the Au_8n+4_(SCH_3_)_4n+8_ quantum-box nanoclusters. Accordingly, for the rational design of high-PLQY Au NCs—especially those targeting efficient near-infrared emission—strategic reduction of structural symmetry, in conjunction with size elongation and AR enhancement, is herein revealed to be an indispensable design principle.

### Fast ISC/RISC Dynamics and Small Singlet–Triplet Gap-Controlled Multi-Emission Mechanism in Quantum-Box Au NCs

Previous studies have shown that both Au_28_(TBBT)_20_ and Au_52_(TBBT)_32_ exhibit dual-emission behavior involving TADF and phosphorescence.^25,26^ To further elucidate the emission characteristics of quantum-box Au NCs, we performed a systematic computational investigation of their excited-state properties and emission mechanisms. In accordance with El-Sayed’s rule, the spin-orbit coupling matrix elements (SOCMEs) between S_1_ and higher-lying triplet states (T_2_, T_3_, and T_4_) are larger than those between S_1_ and the lowest triplet state, T_1_ (Table S4), suggesting that higher-lying triplet states may also contribute to the nonradiative decay pathways of these clusters. Motivated by this observation, we optimized the geometries of the higher-energy triplet states (T_2_, T_3_, and T_4_) and evaluated the adiabatic energy gaps between S_1_ and these triplet states. However, because the PESs lie very close to one another at higher-energy states, stable minima could not be located for some triplet states due to PES crossings. To indirectly evaluate the potential impact of these nonconverged triplet states, we located triplet–triplet conical intersection (CI) geometries along the intersection seam between the crossing triplet potential-energy surfaces and computed the adiabatic energy gaps between S_1_ and these CI geometries. The results show that the energy gaps between S_1_ and higher-lying triplet states (e.g., T_2_) are relatively small, while the corresponding SOCMEs are comparatively large. Together, the combination of small S_1_-T_n_ energy gaps and large SOCMEs suggests that these higher-lying triplet states may participate not only in intersystem crossing (ISC) from S_1_ but also in RISC back to S_1_.

As shown in Figure 4, for Au_36_(SCH_3_)_24_ and Au_52_(SCH_3_)_32_, the higher-lying triplet states (T_2_, T_3_, T_4_) that lie closest in energy to S_1_ could not be fully optimized due to severe PES crossings. Nevertheless, previous studies have shown that these higher-lying triplet states, featuring larger SOCMEs and smaller energy gaps relative to S_1_, can provide more efficient pathways for population transfer from S_1_ to T_1_ via higher triplet intermediates (i.e., S_1_ → T_n_ → T_1_). Notably, all investigated clusters exhibit a relatively small S_1_–T_1_ energy gap, Δ*E*_ST_, consistent with recent reports by Sun and coworkers.^34^ Except for Au_36_(SCH_3_)_24_, Δ*E*_ST_ decreases gradually with increasing cluster size.

**Figure 4:**
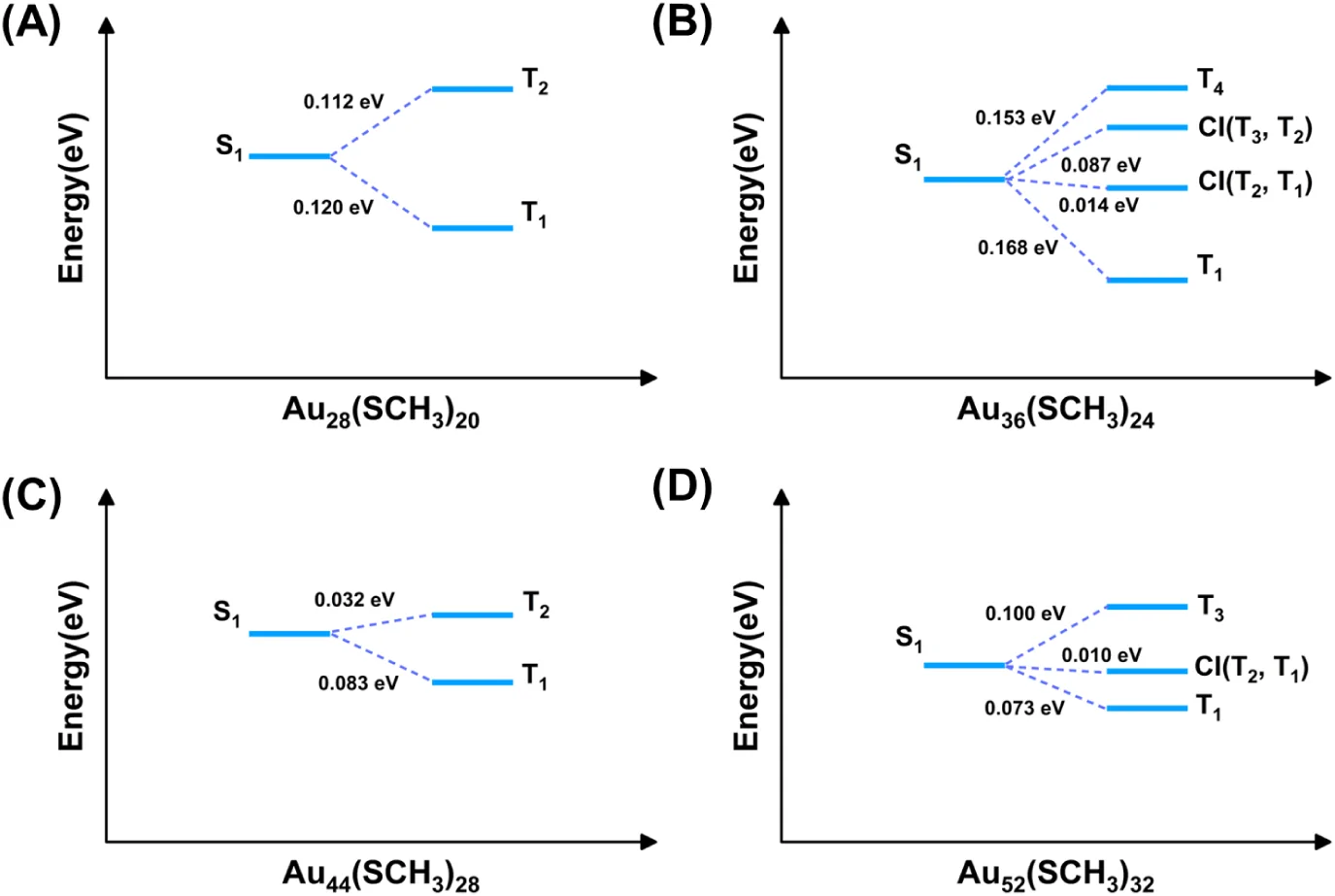
Energy-level diagrams of the lowest singlet (S_1_) and triplet (T_1_, T_2_, T_3_, T_4_) excited states for (A) Au_28_(SCH_3_)_20_, (B) Au_36_(SCH_3_)_24_, (C) Au_44_(SCH_3_)_28_, and (D) Au_52_(SCH_3_)_32_. The energy gaps (Δ*E*_ST_) between S_1_ and the nearest triplet states are indicated. For Au_36_(SCH_3_)_24_ and Au_52_(SCH_3_)_32_, CI between adjacent triplet states (T_3_/T_2_ and T_2_/T_1_) are shown, highlighting possible internal conversion channels within the triplet states.

Owing to the small Δ*E*_ST_ values and relatively large SOCMEs, the S_1_ → T_1_ ISC rates and the T_1_ → S_1_ RISC rates are both large and comparable in magnitude (Table 2), indicating that rapid interconversion between S_1_ and T_1_ is both energetically and kinetically favorable. On this basis, we constructed a three-state kinetic model comprising S_1_, T_1_, and S_0_. By incorporating the calculated ISC, RISC, and radiative rate constants, we simulated the excited-state population dynamics for each cluster (Figure 5). The simulations reveal the coexistence of multiple emission channels, including prompt fluorescence (PF), TADF, and phosphorescence. As a side note, temperature may alter the populations of the states and, accordingly, the ratios of the emissions, such as TADF enhancement at higher temperatures but phosphorescence enhancement at lower temperatures.

**Table 2:** Computed Adiabatic Energy Gaps between S_1_ and T_1_ Δ*E*_ST_, Spin–Orbit Coupling Matrix Elements (SOCMEs), S_1_ → T_1_ ISC Rate Constant *k*_ISC_, and T_1_ → S_1_ RISC Rate Constant *k*_RISC_

		S_1_ → T_1_ ISC		T_1_ → S_1_ RISC	
	Δ*E*_ST_ (eV)	SOCME (cm^–1^)	*k* (s^–1^)	SOCME (cm^–1^)	*k* (s^–1^)
Au_28_(SCH_3_)_20_	0.120	8.11	7.6×10^9^	30.79	8.7 × 10^9^
Au_36_(SCH_3_)_24_	0.168	46.54	3.4×10^10^	10.94	6.2 × 10^8^
Au_44_(SCH_3_)_28_	0.083	20.57	2.3×10^11^	22.29	6.9 × 10^10^
Au_52_(SCH_3_)_32_	0.073	22.10	1.8×10^11^	27.42	4.0 × 10^10^

**Figure 5:**
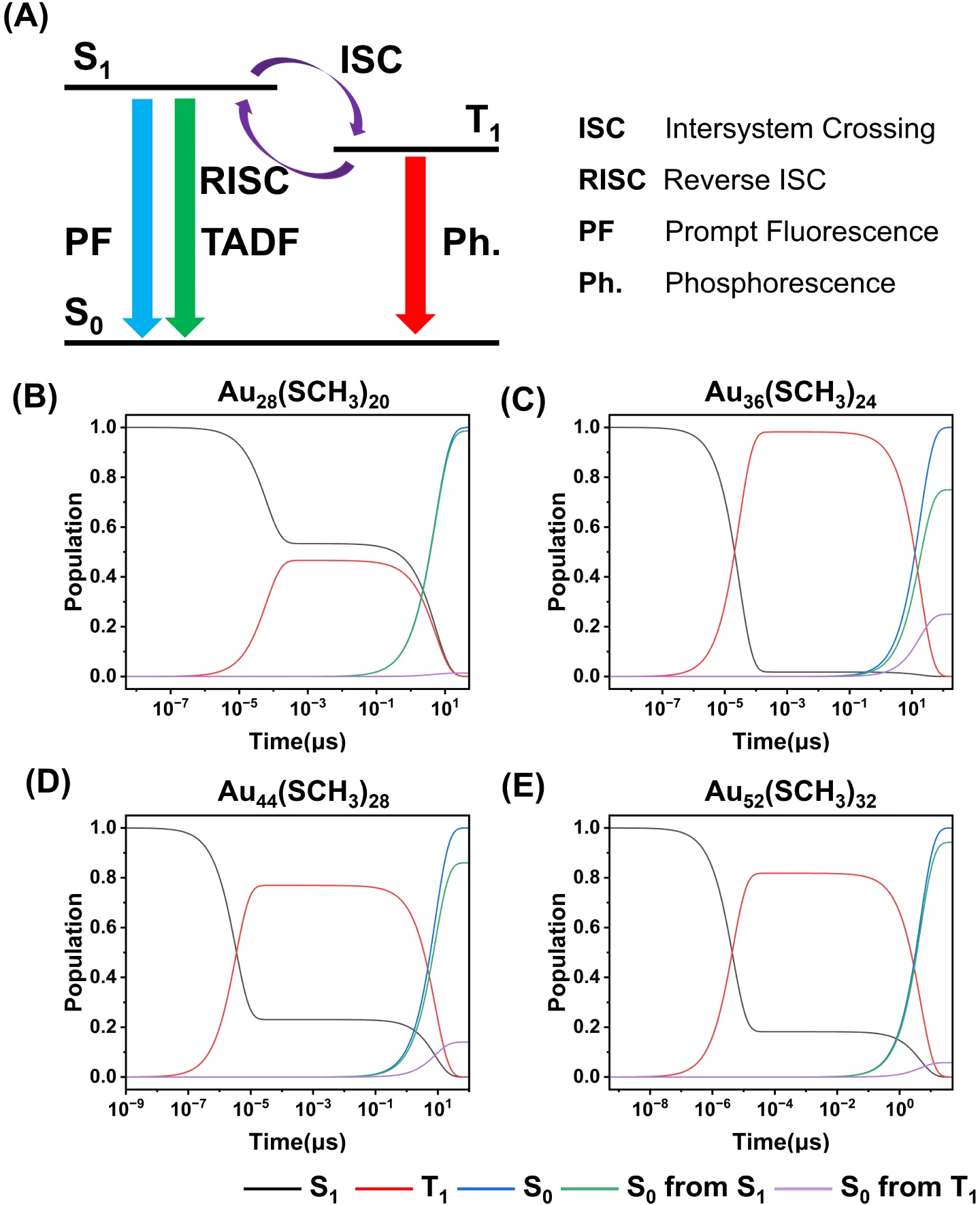
(A) PL mechanisms, and dynamic evolution for (B) Au_28_(SCH_3_)_20_, (C) Au_36_(SCH_3_)_24_, (D) Au_44_(SCH_3_)_28_, and (E) Au_52_(SCH_3_)_32_. The dynamics were simulated at a temperature of 300 K.

For Au_28_(SCH_3_)_20_, the ISC rate constant *k*_ISC_(S_1_ → T_1_) is nearly identical to the RISC rate constant *k*_RISC_(T_1_ → S_1_), enabling a quasi-equilibrium between the S_1_ and T_1_ states. However, because the fluorescence radiative rate constant is approximately two orders of magnitude larger than the phosphorescence radiative rate constant, the equilibrium is slightly shifted toward the S_1_ population, leading to the coexistence of PF and TADF, with only a weak phosphorescence component. Notably, since the ISC rate is much faster than the fluorescence radiative rate, population exchange between S_1_ and T_1_ occurs on the sub-nanosecond timescale. As a result, PF and TADF are difficult to distinguish in conventional microsecond-scale photoluminescence measurements and may require ultrafast time-correlated single-photon counting experiments for unambiguous resolution.

For Au_44_(SCH_3_)_28_ and Au_52_(SCH_3_)_32_, the smaller Δ*E*_ST_ leads to substantially larger *k*_ISC_ and *k*_RISC_, so PF and TADF likewise coexist. Moreover, their increased phosphorescence radiative rates (2.2 × 10^4^ and 1.5 × 10^4^ s^–1^ for Au_44_(SCH_3_)_28_ and Au_52_(SCH_3_)_32_, respectively) introduce a non-negligible phosphorescence component. In contrast, Au_36_(SCH_3_)_24_ exhibits a much larger S_1_–T_1_ SOCME (46.54 cm^–1^ versus 8.11 cm^–1^ for Au_28_(SCH_3_)_20_), enabling rapid ISC, whereas a smaller T_1_–S_1_ SOCME, together with a larger Δ*E*_ST_ (0.168 eV), slows down the RISC process. Consequently, the population is efficiently transferred to, and trapped in, T_1_, suppressing PF and leading to the coexistence of TADF and phosphorescence. Consistently, the temperature-dependent PL spectra exhibit a progressive redshift of the emission peaks upon cooling, further supporting the coexistence of TADF and phosphorescent emission channels. The multi-emission nature is further supported by experimental observations of at least two PL lifetimes (see next Section), with one on the order of tens of nanoseconds (assigned to TADF) and the other on the order of hundreds of nanoseconds (assigned to phosphorescence).

In summary, owing to their small Δ*E*_ST_ and large SOCMEs, quantum-box Au NCs exhibit ISC and RISC rate constants that are much larger than their radiative rate constants, leading to rapid S_1_–T_1_ interconversion and establishing a dynamic equilibrium between these two states. Because fluorescence radiative rates are generally higher than phosphorescence radiative rates, the steady-state population is typically biased toward S_1_, so emission often contains a substantial fluorescence-derived contribution while simultaneously allowing multiple channels such as PF, TADF, and phosphorescence to coexist. Importantly, the dual-emission behavior of quantum-rod clusters (e.g., Au_42_(SCH_3_)_32_) arises from a different mechanism: previous computations by Cui and coworkers attributed the dual emission of Au_42_(SCH_3_)_32_ to its unusually large fluorescence radiative rate, which can effectively compete with ISC,^30^ while its relatively large Δ*E*_ST_ suppresses RISC and thus disfavors TADF. Consistent with recent studies on high AR quantum-rod clusters (e.g., Au_42_(SCH_3_)_32_ and Au_60_(SCH_3_)_44_), this contrast highlights that quantum-box clusters, with much smaller transition dipole moments and correspondingly lower fluorescence radiative rates, exhibit dual emission primarily enabled by fast ISC/RISC dynamics at small Δ*E*_ST_, whereas quantum-rod clusters’ strong fluorescence involves a kinetic competition with ISC under larger Δ*E*_ST_.

In recent years, various Au NCs displaying multi-emission (including dual-emission) behavior have been reported experimentally. However, their underlying photophysical mechanisms differ markedly. The distinct multi-emission mechanisms observed in these two classes of clusters (quantum box vs. quantum rod) can primarily be traced to their different Δ*E*_ST_ values, a phenomenon that merits further systematic investigation.

To minimize the influence of S_1_ and T_1_ structural relaxation on Δ*E*_ST_ and to reveal the intrinsic electronic structure origin of this difference, we calculated the vertical S_1_–T_1_ energy gaps, Δ*E*_v_(S_1_, T_1_), based on the optimized ground-state S_0_ geometries of these clusters. As shown in Table 3, the quantum box clusters exhibit relatively small Δ*E*_v_(S_1_, T_1_) values. The discrepancies between these vertical gaps and the corresponding adiabatic gaps can be rationalized by the excited-state structural relaxation in the S_1_ and T_1_ states.

**Table 3:** Calculated Energy Gaps, Transition Characteristics, and Charge Separation Descriptors for the Clusters^a^

			S_0_ → S_1_		S_0_ → T_1_	
	Δ*E*_ST_ (eV)	Δ*E*_v_(S_1_, T_1_) (eV)	D index (Å)	S_r_	D index (Å)	S_r_
Au_28_(SCH_3_)_20_	0.120	0.159	0.719	0.662	0.619	0.688
Au_36_(SCH_3_)_24_	0.168	0.165	0.215	0.663	0.298	0.769
Au_44_(SCH_3_)_28_	0.083	0.119	0.333	0.721	0.222	0.761
Au_52_(SCH_3_)_32_	0.073	0.139	0.592	0.670	0.120	0.726

a Δ*E*_ST_ and Δ*E*_v_(S_1_, T_1_) represent the adiabatic and vertical singlet–triplet energy gaps, respectively. The D index quantifies the electron–hole separation distance, and S_r_ denotes the electron–hole overlap strength.

Within a simplified HOMO–LUMO excitation picture, the S_1_ and T_1_ states can be viewed as arising predominantly from promotion of an electron from the ground-state HOMO to the LUMO, leaving a hole in the HOMO. The key distinction between S_1_ and T_1_ states lies in the spin configuration of the electron–hole pair, namely antiparallel spins in S_1_ and parallel spins in T_1_. The exchange energy, K, governs the vertical energy gap between S_1_ and T_1_, which can be approximated as Δ*E*_v_(S_1_, T_1_) ≈ 2K. Based on this relationship, we constructed real-space maps of the exchange-energy contribution K for the quantum-box clusters and the quantum-rod Au_42_(SCH_3_)_32_ cluster (Figure S4). Notably, Au_42_(SCH_3_)_32_, which has been reported to exhibit a relatively large Δ*E*_ST_, shows a more spatially extended distribution of exchange-energy density, whereas the quantum-box clusters display a more localized and weaker distribution. Since K is closely related to, and approximately scales with, the spatial overlap between the HOMO and LUMO, reducing the electron–hole spatial overlap is crucial for achieving a small intrinsic Δ*E*_v_(S_1_, T_1_).

Furthermore, the electron–hole overlap index (S_r_) from the electron–hole analysis provides a quantitative explanation for the differences in Δ*E*_v_(S_1_, T_1_) among these clusters. Previous calculations show that Au_42_(SCH_3_)_32_ and Au_60_(SCH_3_)_44_, which possess relatively large Δ*E*_ST_, exhibit high S_r_ values of 0.78 and 0.79,^31^ respectively, indicating that the excited-state electron and hole are confined along a quasi-one-dimensional direction with substantial spatial overlap, consistent with the corresponding electron–hole pair distributions. In contrast, the quantum-box clusters, characterized by smaller ARs, allow the excited-state electron and hole to delocalize along different directions in three-dimensional space (Figure S5), leading to significantly reduced spatial overlap. Consequently, Au_28_(SCH_3_)_20_, Au_36_(SCH_3_)_24_, Au_44_(SCH_3_)_28_, and Au_52_(SCH_3_)_32_ exhibit smaller S_r_ values and correspondingly smaller Δ*E*_v_(S_1_, T_1_) (Table 3), which highlight an intrinsic electronic-structure origin for their reduced singlet–triplet splitting.

Notably, the relationship between structure and Δ*E*_v_(S_1_, T_1_) discussed above, namely that spatial separation of electron–hole pairs leads to a reduced Δ*E*_v_(S_1_, T_1_), is different from that in conventional organic TADF molecules.^35,36^ In organic conjugated systems, highly planar π-conjugated backbones typically promote strong spatial overlap between electrons and holes, resulting in large exchange energy, K, and making it difficult to achieve a small Δ*E*_v_(S_1_, T_1_). Accordingly, structural strategies such as introducing twisted donor–acceptor (D-A) architectures (Figure 6A) to reduce electron–hole overlap, or constructing B/N co-doped aromatic frameworks (Figure 6B)^37–39^ to generate spatially alternating yet overall weak electron–hole overlap, are commonly employed. In contrast, ligand-protected Au NCs, especially those with larger sizes and core–shell structures, provide more diverse pathways for electron–hole separation. In these systems, electron–hole pairs can be spatially separated not only via core–shell charge-transfer (CT) states but also through geometric and dimensional effects. For example, in clusters with relatively small ARs, such as the three-dimensional Au_8n+4_(SCH_3_)_4n+8_ quantum-box series (Figure 6D), the excited-state electron and hole can delocalize along different directions in three-dimensional space, substantially reducing their spatial overlap and thereby leading to a small Δ*E*_v_(S_1_, T_1_). By contrast, in much elongated quantum-rod clusters such as Au_42_(SCH_3_)_32_ and Au_60_(SCH_3_)_44_, the electron and hole are largely confined along a one-dimensional axis (Figure 6C), resulting in larger spatial overlap and consequently larger Δ*E*_v_(S_1_, T_1_).

**Figure 6:**
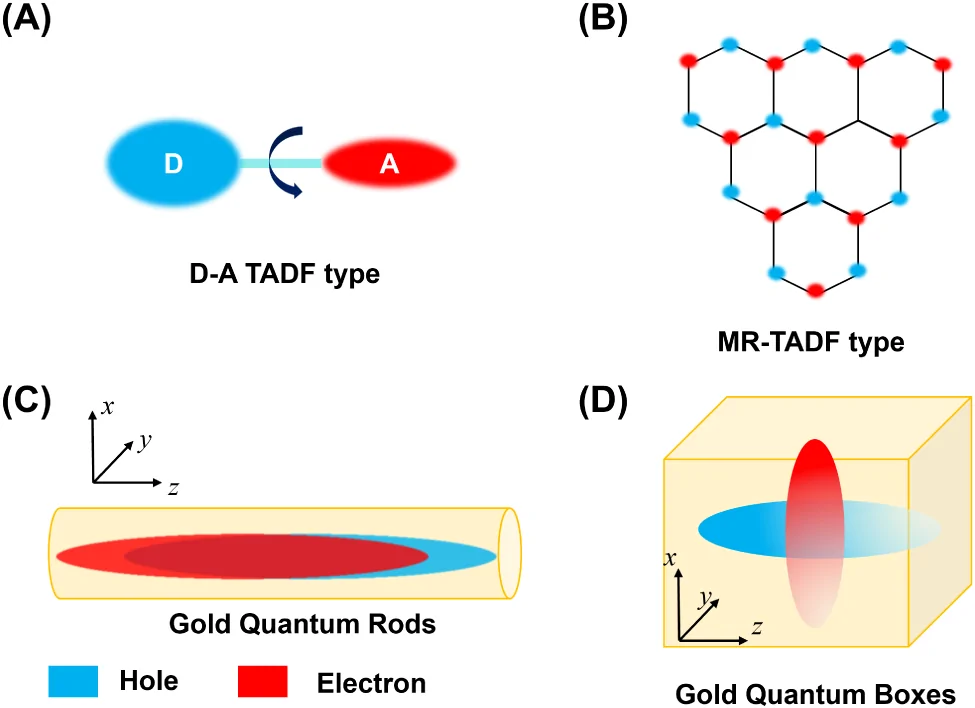
Schematic illustrations of different TADF systems and quantum-confined structures. (A) Donor–acceptor (D–A)-type TADF mechanism. (B) Multiple-resonance (MR) TADF framework with alternating electron-donating and -accepting sites. (C) Gold quantum rods showing spatial overlap of hole and electron distributions along the longitudinal axis. (D) Gold quantum boxes illustrating confined electron and hole distributions in three dimensions.

### Ligand-Mediated Nonradiative Decay: Low-Frequency Vibrations and Size-Dependent Relaxation Dynamics in Quantum-Box Au NCs

Given that the quantum-box clusters predominantly undergo deactivation from the S_1_ state to the ground state, we next calculated the S_1_ → S_0_ internal conversion (IC) rate constants *k*_IC_(S_1_ → S_0_) and performed detailed Huang-Rhys factor and reorganization energy decomposition analyses. The computed IC rate constants confirm that nonradiative S_1_ → S_0_ IC dominates the excited-state relaxation pathways, which is consistent with the experimentally observed low PLQYs (Table 4). It should be noted that the calculated *k*_IC_(S_1_ → S_0_) values are likely overestimated due to the neglect of anharmonic effects, which may lead to underestimated excited-state lifetimes and PLQY values relative to experimental measurements. Nevertheless, the overall theoretical trends in excited-state lifetimes and PLQYs agree well with experimental observations. Specifically, Au_36_(SCH_3_)_24_ and Au_52_(SCH_3_)_32_ exhibit higher PLQYs within the series due to their larger radiative decay rates, whereas Au_28_(SCH_3_)_20_ and Au_44_(SCH_3_)_28_ display lower (Table 4).

**Table 4:** Computed Radiative Rate Constants *k*_r_, Internal Conversion Rate Constants *k*_IC_ at the Optimized S_1_ Structure, Theoretical Excited-State Lifetimes *τ*_theo_ and PLQYs, and Experimental Excited-State Lifetimes *τ*_exp_ and PLQY Values

	Au_28_(SCH_3_)_20_	Au_36_(SCH_3_)_24_	Au_44_(SCH_3_)_28_	Au_52_(SCH_3_)_32_
*k*_r_(S_1_ → S_0_) (s^–1^)	3.5 × 10^5^	2.3 × 10^6^	4.5 × 10^5^	1.1 × 10^6^
*k*_IC_(S_1_ → S_0_) (s^–1^)	6.0 × 10^9^	4.6 × 10^9^	8.4 × 10^9^	3.3 × 10^9^
*τ*_theo_ (ns)	0.167	0.210	0.119	0.306
*τ*_exp_ (ns)^a^	68	93	115	135
*τ*_exp_ (ns)^b^	τ_1_ = 51 (A_1_ = 70%),	τ_1_ = 105 (A_1_ = 81%),	τ_1_ = 61 (A_1_ = 82%),	τ_1_ = 36 (A_1_ = 16%),
	τ_2_ = 605 (A_2_ = 30%)	τ_2_ = 700 (A_2_ = 19%)	τ_2_ = 855 (A_2_ = 18%)	τ_2_ = 153 (A_2_ = 84%)
	(τ_av_ = 217)	(τ_av_ = 218)	(τ_av_ = 204)	(τ_av_ = 134)
Cal. PLQY	0.0058%	0.05%	0.0054%	0.034%
Exp. PLQY	≈0.16%	≈0.7%	≈0.27%	≈3.8%

a Measured by transient absorption at room temperature and ambient conditions.

b Measured by time-resolved PL at r.t. and ambient conditions, τ_av_ $=\sum_{n}\tau_{n}A_{n}$ (where A_n_ values are the percentages of photons corresponding to τ_n_). All experiments refer to the TBBT-protected NCs.

Huang–Rhys factor and reorganization energy analyses (Figures S6 and S8) reveal that low-frequency vibrational modes play a decisive role in governing the excited-state dynamics of these clusters, in good agreement with our previous investigations on Au_38_S_2_(SR)_20_ and Au_25_(SR)_18_.^40,41^ Ligand-protected Au NCs typically adopt a well-defined core–shell architecture (the shell refers to the staple-like surface motifs), which gives rise to a dense manifold of vibrational modes, especially in the low-frequency region. In the low-frequency regime, vibrational modes arise from both the gold core and the protecting ligand shell. The gold core contributes global breathing modes and local Au–Au stretching motions, whereas the ligand shell supports collective vibrations of Au_*x*_–S_*x*+1_ staple motifs (x = 1, 2, etc.) as well as torsional and librational modes of the organic tails (−R groups). Moreover, these vibrational modes are often strongly coupled, further complicating the vibrational density of states in ligand-protected Au NCs.

Experimentally, resolving such low-frequency modes at the atomic level remain highly challenging. Meanwhile, theoretical studies often employ simplified ligand models to reduce computational costs, which can inadvertently underestimate the ligand contribution to nonradiative relaxation. As a result, prior work has frequently attributed the low-frequency vibrational modes of ligand-protected Au NCs primarily to motions of the metal core.^42–44^ Our current work instead reveals the critical contributions from the low-frequency modes of surface staples and organic tails. To explicitly quantify the contributions of distinct vibrational modes to nonradiative relaxation, we decomposed the Huang–Rhys factors and reorganization energies according to the structural components of the clusters (Figure 7 and Figure S7). The results clearly demonstrate that low-frequency vibrational modes associated with methyl groups (torsional and librational motions) provide the dominant contributions to both the Huang–Rhys factors and the total reorganization energies, whereas the contribution from the gold core is comparatively minor.

**Figure 7:**
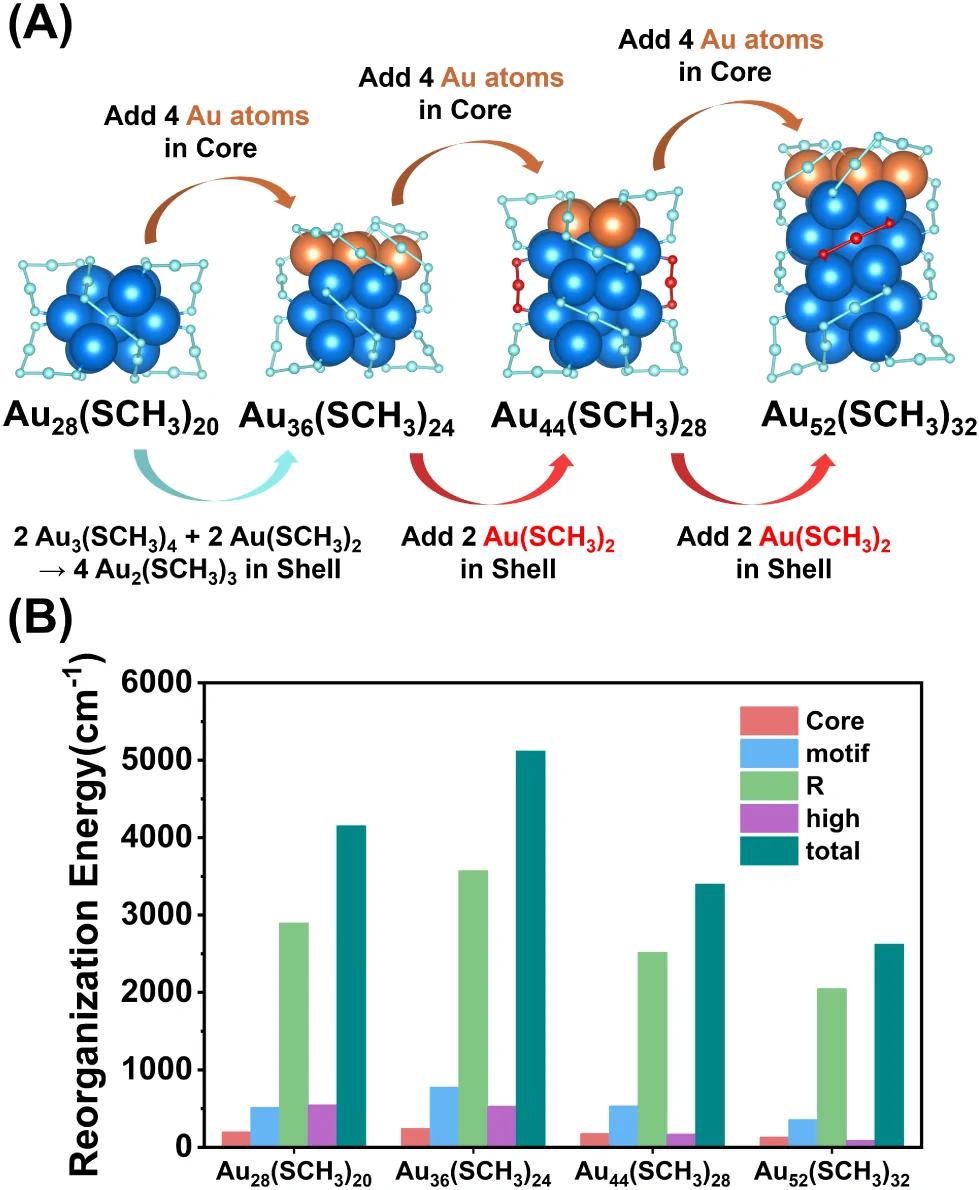
(A) Schematic growth pathway from Au_28_(SCH_3_)_20_ to Au_52_(SCH_3_)_32_, where each transformation proceeds via the addition of 4 Au atoms to the Au core (orange) and incorporation of Au­(SCH_3_)_2_ staples (red) into the ligand shell. Blue spheres denote preexisting core atoms, and cyan frameworks indicate surface motif staples. (B) Decomposition of the reorganization energy into contributions from different vibrational mode groups for Au_8n+4_(SCH_3_)_4n+8_. The vibrational modes are classified into core modes, R (R = CH_3_) ligand modes, motif staple modes, and high-frequency modes.

Notably, the total reorganization energy, as well as the individual contributions from methyl-group vibrations and staple-motif vibrations, exhibits an interesting nonmonotonic size dependence: it increases from Au_28_(SCH_3_)_20_ to Au_36_(SCH_3_)_24_ and then decreases for Au_44_(SCH_3_)_28_ and Au_52_(SCH_3_)_32_. For high-frequency modes above 500 cm^–1^, such as S–C and C–H stretching vibrations, their contributions to the Huang–Rhys factors are negligible, while the corresponding contributions to the reorganization energy gradually decrease with increasing cluster nuclearity. Consistent with this trend, Au_28_(SCH_3_)_20_ to Au_36_(SCH_3_)_24_ exhibit relatively large root-mean-square deviation (RMSD) values (0.349 and 0.367 Å, respectively) following excited-state structural relaxation, indicating more pronounced geometric distortions. In contrast, Au_44_(SCH_3_)_28_ and Au_52_(SCH_3_)_32_ display smaller RMSD values (0.242 and 0.244 Å, respectively) and smaller structural deformations, again manifesting an interesting nonmonotonic size-dependent behavior. To further elucidate the structural origin of these variations, we decomposed the total RMSD into contributions from the metal core, staple motifs, and terminal CH_3_ groups (Table S5). The terminal CH_3_ groups exhibit the largest structural changes, followed by the staple motifs, whereas the metal cores undergo the smallest structural changes. This indicates that the structural reorganization associated with excited-state relaxation occurs predominantly in the peripheral ligand region, particularly in the terminal CH_3_ groups, while the overall metal-core framework remains relatively stable.

To rationalize these observations, we further analyzed the structural growth pattern of the quantum-box clusters (Figure 7A) and correlated it with the decomposed reorganization energies (Figure 7B). The gold cores expand layer-by-layer via the addition of four Au atoms at each growth step, whereas the evolution of the outer staple motifs differs drastically. Because low-frequency motions of the ligands and staple motifs dominate the total reorganization energy, the ligand- and motif-derived contributions serve as a direct structural-dynamical descriptor for shell-mediated nonradiative energy dissipation. From Au_36_(SCH_3_)_24_ to Au_44_(SCH_3_)_28_ and Au_52_(SCH_3_)_32_, each four-Au-atom core expansion is accompanied by the incorporation of two primary staple motifs, Au­(SCH_3_)_2_, into the ligand shell. Notably, these newly formed primary staples are predominantly located in the central longitudinal (midsection) region of the ligand shell. This structural evolution increases the ligand packing density in the midsection, thereby restricting the torsional and librational motions of the R groups as well as the vibrations of the staple motifs. Consistently, both the R-group and staple-motif contributions to the reorganization energy decrease from Au_36_(SCH_3_)_24_ to Au_44_(SCH_3_)_28_ and further to Au_52_(SCH_3_)_32_, leading to a pronounced reduction in the total reorganization energy and indicating suppressed ligand-mediated nonradiative relaxation.

By contrast, during the growth from Au_28_(SCH_3_)_20_ to Au_36_(SCH_3_)_24_, although the core likewise expands by four Au atoms, two long tertiary staple motifs, Au_3_(SCH_3_)_4_, are converted into four secondary staples, Au_2_(SCH_3_)_3_, at the relatively open termini of the clusters. Because this transformation does not substantially compress the terminal ligand arrangement, the ligands at the ends remain relatively unconstrained and can undergo larger-amplitude low-frequency motions. In agreement with this picture, the R component (and thus the total) reorganization energy reaches a maximum at Au_36_(SCH_3_)_24_, implying enhanced ligand-mediated nonradiative dissipation. Therefore, along the one-dimensional structural evolution of the quantum box clusters, variations in the structure of the outer staple motifs give rise to an interesting nonmonotonic trend in ligand-dominated nonradiative relaxation: ligand-induced nonradiative relaxation increases from Au_28_(SCH_3_)_20_ to Au_36_(SCH_3_)_24_ and subsequently decreases for Au_44_(SCH_3_)_28_ and Au_52_(SCH_3_)_32_.

It should be noted that to reduce computational cost, the simplification of the R groups (−SCH_3_ substitution for −S–Ph–^*t*^Bu) in our calculations precludes an explicit description of vibrational contributions from the relatively rigid phenyl rings. Nevertheless, larger R groups are likely to further increase the ligand-shell compactness in the longitudinal (midsection) region, although the extent may depend on steric matching and packing geometry. Recent studies reporting a pronounced enhancement of PLQY upon R-group modification in clusters such as Au_52_(SCH_3_)_32_ are consistent with a critical role of R groups in regulating nonradiative processes, potentially by restricting low-frequency ligand motions.^26^ Taken together, these considerations suggest that reducing the number of terminal SCH_3_ groups in S–Ph–^*t*^Bu ligands could, if steric hindrance is relieved, strengthen π–π stacking interactions between phenyl rings, suppress low-frequency vibrational modes of the aromatic units, and thereby inhibit nonradiative decay and enhance PLQY. Moreover, such a modification may also reduce the contribution of low-frequency vibrations from more peripheral SR groups to nonradiative relaxation, manifesting as a decreased ligand contribution to the reorganization energy. Therefore, rather than cluster size alone, the longitudinal compactness of the ligand shell induced by one-dimensional growth is more likely to play a dominant role in suppressing low-frequency vibrations and reducing nonradiative losses.

Unlike the Au_8n+4_(SCH_3_)_4n+8_ quantum-box clusters, in which longer staple motifs are prevalent, representative quantum-rod clusters such as Au_42_(SCH_3_)_32_ exhibit a higher proportion of primary staple motifs (i.e., Au­(SCH_3_)_2_ surface units). In this comparison, the much larger AR is accompanied by an increased number of primary staples, which can promote a more compact ligand arrangement in the central longitudinal (midsection) region and thus suppress low-frequency ligand motions. This interpretation is consistent with our earlier analysis that reduced ligand-/motif-dominated low-frequency contributions (e.g., decreased ligand and motif components of the reorganization energy) correlate with diminished ligand-mediated nonradiative dissipation. Beyond increasing the fraction of primary staples and reducing long staple motifs, tailoring the spatial distribution of staple motifs to strengthen interligand interactions (e.g., π–π/van der Waals packing) also offers a viable route for inhibiting nonradiative processes.

In summary, the smaller ARs and higher symmetry of quantum box clusters restrict the magnitude of transition dipole moments, resulting in intrinsically low radiative rates. Under symmetry-imposed constraints, the transition dipole moment does not increase monotonically with cluster size or ARs; instead, it exhibits oscillatory behavior, consistent with the fluctuations observed during the structural evolution. For nonradiative processes, low-frequency vibrational modes associated with surface ligands provide efficient channels for excited-state energy dissipation and thus strongly facilitate nonradiative decay. During the size evolution, the quantum box clusters display a nonmonotonic trend in ligand-dominated nonradiative relaxation: an initial increase from Au_28_(SCH_3_)_20_ to Au_36_(SCH_3_)_24_, followed by a decrease from Au_36_(SCH_3_)_24_ to Au_44_(SCH_3_)_28_ to Au_52_(SCH_3_)_32_. This interesting nonmonotonic behavior arises from the progressively enhanced longitudinal (midsection) compactness of the ligand shell induced by the increasing incorporation of primary staple motifs. Compared with quantum rod clusters, which typically contain a high proportion of primary staples, quantum box clusters possess fewer primary staples even in the largest Au_52_(SCH_3_)_32_ cluster because of geometric constraints. Consequently, owing to their higher symmetry and smaller ARs, quantum box clusters exhibit relatively low radiative rates, and ligand-mediated nonradiative decay is not effectively suppressed, collectively leading to their universally low PLQYs.

## Conclusions

This work clarifies the intrinsic structure-luminescence correlation within Au_8n+4_(TBBT)_4n+8_ quantum box nanoclusters and establishes a unified theoretical paradigm to rationalize their low PLQYs and dual/multiple emission behavior via theoretical calculations. The intrinsically low PLQYs of this series arise from the combined effects of radiative and nonradiative processes. The high quasi-symmetry of these clusters reduces transition dipole moments and suppresses radiative decay. Meanwhile, abundant low-frequency vibrational modes from peripheral thiolate ligands and staple motifs facilitate excited-state nonradiative relaxation, further lowering PLQYs. Their moderate aspect ratios induce small singlet–triplet energy gaps, enabling the coexistence of prompt fluorescence, thermally activated delayed fluorescence, and phosphorescence emission channels.

These fundamental insights not only deepen the mechanistic understanding of excited-state photophysics in ligand-protected Au NCs but also deliver rational design guidelines for constructing high-PLQY counterparts via precise modulation of cluster size, aspect ratio, and ligand-shell architecture. Based on the revealed structure–property relationships, we further propose feasible structural regulation strategies: reducing the overall point-group symmetry, enlarging the geometric aspect ratio, and strengthening ligand rigidity as well as interligand intermolecular interactions to suppress low-frequency vibrational motions. Such strategies are anticipated to simultaneously red-shift emission into the NIR window, suppress nonradiative energy loss, and remarkably boost PLQY. The fundamental design principles established herein offer clear structure–property benchmarks for the rational fabrication of high-PLQY, NIR-emissive ligand-protected Au NCs and lay a solid theoretical basis for future PL performance optimization through sophisticated ligand engineering and structural tailoring.

### Simulation Methods

All DFT and TD-DFT calculations were performed using the ORCA 5.0.2 package.^45–47^ The PBE0 hybrid functional^48^ in combination with the def2-SV­(P) basis set, was employed for ground- and excited-state geometry optimizations of the Au NCs, as well as for the search for conical intersection (CI) structures. To improve computational efficiency, the resolution-of-identity (RI) approximation was applied for both DFT and TD-DFT calculations, with the def2/J auxiliary basis set.^49^ All structures were optimized in the gas phase. The convergence criteria for geometry optimizations were set to 1.0 × 10^–6^ Hartree for the total energy and 3.0 × 10^–4^ Hartree/Å for the maximum gradient. Geometry optimizations of the ground singlet state S_0_ and the lowest triplet state T_1_ were carried out at the DFT level, whereas the first singlet excited state S_1_ and higher-lying triplet excited states (T_2_, T_3_, and T_4_) were optimized using TD-DFT. Vibrational frequency calculations for both ground and excited states were performed at the same level of theory (PBE0/def2-SV­(P)) using the Gaussian 16 program.^50^ SOCMEs were calculated using ORCA 5.0.2 with the PBE0 functional. The DKH-def2-SVP basis set was applied to S, C, and H atoms, while the SARC-DKH-SVP basis set was used for Au atoms. The radiative rate constant was calculated using Einstein’s radiation rate constant equation:

1 $$k_{r}=\frac{2}{3}fE^{2}$$

where *k_r_* is the fluorescence radiative rate constant, *f* is the oscillator strength, *E* is the vertical emission energy. For the phosphorescence radiative rate constant, the oscillator strength was obtained by calculating the SOCMEs based on the optimized T_1_ structure.

On the basis of the calculated vibrational frequencies and SOCMEs, IC and ISC rate constants were evaluated using the FCclasses3 program,^51^ enabling a quantitative analysis of nonradiative decay processes. Huang–Rhys factors and reorganization energy decompositions were also obtained from FCclasses3. Electron–hole analyses^52^ were performed, and cube files of the frontier molecular orbitals were generated using the Multiwfn 3.8 (dev) program^53^ for subsequent visualization. These data were further used to construct spatial distributions of transition dipole moment density (TDMD) and exchange energy densities, providing an intuitive visualization of the excited-state electronic structures and their correlation with cluster symmetry. In addition, based on the Huang–Rhys factors and reorganization energies obtained from FCclasses3, a decomposition analysis was performed to quantify the contributions from different structural units. To reduce computational cost, the thiolate ligand substituents in Au_28_(TBBT)_20_, Au_36_(TBBT)_24_, Au_44_(TBBT)_28_, and Au_52_(TBBT)_32_ were simplified to SCH_3_.

## Experimental Section

The Au_28_(TBBT)_20_, Au_36_(TBBT)_24_, Au_44_(TBBT)_28_, and Au_52_(TBBT)_32_ NCs^15^ were synthesized at room temperature, and the products were separated by thin-layer chromatography (TLC; developing solvent: 2:5 (v/v) DCM:n-hexane), with additional TLC purification as needed. In a typical procedure, a 50 mL round-bottom flask was charged with HAuCl_4_·3H_2_O (78.74 mg, 0.2 mmol). Under rapid stirring (550 rpm) with a stir bar, 15 mL of tetrahydrofuran containing trioctylammonium bromide (136.7 mg, 0.25 mmol) was added. The solution turned deep orange within 20 min. 4-tert-butylbenzenethiol (133 μL, 0.8 mmol) was then introduced. After 30 min, the mixture became light yellow, indicating the formation of Au­(I)-SR complexes. Subsequently, a freshly made, ice-cold NaBH_4_ solution (38 mg, 1 mmol) was quickly added. The mixture turned black, and the reaction was allowed to proceed for 1 h. The aqueous layer was then removed, and the organic phase was concentrated to ∼3 mL under reduced pressure. The organic layer was washed five times with 12 mL of methanol to remove residual thiol. For the second step, 200 μL 4-tert-butylbenzenethiol was added to the organic phase, which was dissolved in 2 mL of toluene. The mixture was heated to 50 °C and kept at this temperature in air with gentle stirring (∼60 rpm) for 16 h. The product was then separated from the thiol/toluene mixture by washing with excess methanol. The remaining dark material was extracted with a minimal amount of dichloromethane and then purified by TLC.

The photoluminescence (PL) spectra of NCs in toluene at room temperature were collected on an FLS1000 spectrofluorometer (Edinburgh Instruments) equipped with a broadband detector (600–1700 nm, cooled to −80 °C with liquid nitrogen). The PL lifetimes were measured by two methods: time-correlated single-photon counting and multi-channel scaling (MCS) photon counting, and the sample was excited at 450 nm with an EPL-450 picosecond pulsed diode laser (∼80 ps; Edinburgh Instruments). Cryogenic PL was performed on a home-built, low-temperature system comprising the FLS1000 spectrofluorometer, a vacuum pump, an Optistat CF2 cryostat (Oxford Instruments), and a temperature controller; liquid nitrogen was used as the cryogen. For cryogenic PL, the NCs were dissolved in 2-methyltetrahydrofuran, a cryogenic solvent that forms a clear glass at cryogenic temperatures.

## Supplementary Material



## Data Availability

All the data are included in the article and/or the Supporting Information.

## Notes

Includes the character tables for different point groups, computed spin–orbit coupling matrix elements (SOCMEs), the root mean square deviation (RMSD) values, the isosurfaces of the exchange-energy densities, the electron-hole pair distributions, Huang-Rhys factors, the reorganization energy contributions from each normal mode, Supporting Tables S1-S5 and Supporting Figures S1–S8 (PDF)
