# Supplementary material for: Mechanism of Multiple Emission in Gold Quantum Boxes and Design Rules for Strong Near-Infrared Luminescence
Source: ACS Nano. 2026 Sep 22;20(39):26599–611. doi: 10.1021/acsnano.6c13094 (PMC13648264; doi:10.1021/acsnano.6c13094)
Supplement: Supplementary file 1 [file nn6c13094_si_001.pdf]

Supporting Information

# Mechanism of Multiple Emission in Gold Quantum Boxes and Design Rules for Strong Near-Infrared Luminescence

Kang Li[1], Yitong Wang,[3] Pu Wang*,[1], Rongchao Jin*,[3], and Yong Pei*,[1,2]

[1] Department of Chemistry, Key Laboratory of Environmentally Friendly Chemistry and Applications of Ministry of Education, Xiangtan University, Hunan Province 411105, China

[2] College of Chemistry & Chemical Engineering, Hunan Normal University, Changsha, Hunan 410081, China

[3] Department of Chemistry, Carnegie Mellon University, Pittsburgh, Pennsylvania 15213, United State

**Corresponding Authors**

*E-mail: 90wangpu@xtu.edu.cn (P. W.); rbj5326@psu.edu (R. J.); ypei2@xtu.edu.cn (Y. P.)

**Table S1.** The character table for point group $D_2$, SBF is the symmetrized basis function.

| $D_2$ | $E$ | $C_2(z)$ | $C_2(y)$ | $C_2(x)$ | SBF |
|---|---|---|---|---|---|
| **A** | 1 | 1 | 1 | 1 | $x^2, y^2, z^2$ |
| $B_1$ | 1 | 1 | -1 | -1 | $z$ |
| $B_2$ | 1 | -1 | 1 | -1 | $y$ |
| $B_3$ | 1 | -1 | -1 | 1 | $x$ |

**Table S2.** The character table for point group $D_{2d}$, SBF is the symmetrized basis function.

| $D_{2d}$ | $E$ | $2C_2(z)$ | $2C'_2$ | $2S_4$ | $2\sigma_d$ | SBF |
|---|---|---|---|---|---|---|
| $A_1$ | 1 | 1 | 1 | 1 | 1 | $z^2, x^2 + y^2$ |
| $A_2$ | 1 | 1 | -1 | 1 | -1 | $R_z$ |
| $B_1$ | 1 | -1 | 1 | -1 | 1 | $x^2 - y^2$ |
| $B_2$ | 1 | -1 | -1 | -1 | -1 | $xy$ |
| **E** | 2 | -2 | 0 | 0 | 0 | $(x, y), (R_x, R_y)$ |

**Table S3.** The character table for point group $C_i$, SBF is the symmetrized basis function.

| $C_i$ | $E$ | $i$ | SBF |
|---|---|---|---|
| $A_g$ | 1 | 1 | $x^2, y^2, z^2$ |
| $A_u$ | 1 | -1 | $x, y, z$ |

**Table S4.** Computed Spin−Orbit Coupling Matrix Elements (SOCMEs).

| | SOCME ($cm^{-1}$) | | | |
|---|---|---|---|---|
| | $S_1 \rightarrow T_1$ | $S_1 \rightarrow T_2$ | $S_1 \rightarrow T_3$ | $S_1 \rightarrow T_4$ |
| $Au_{28}(SCH_3)_{20}$ | 8.11 | 222.85 | 261.16 | 297.57 |
| $Au_{36}(SCH_3)_{24}$ | 46.54 | 130.90 | 221.65 | 76.58 |
| $Au_{44}(SCH_3)_{28}$ | 20.57 | 98.99 | 210.56 | 158.01 |
| $Au_{52}(SCH_3)_{32}$ | 22.10 | 69.77 | 238.19 | 97.90 |

**Table S5.** Root-Mean-Square Deviation (RMSD, Å) of the Optimized Excited-State Structures ($S_1$ and $T_1$) Relative to the Ground-State Structure ($S_0$) of $Au_{28}(SCH_3)_{20}$, $Au_{28}(SCH_3)_{24}$, $Au_{28}(SCH_3)_{28}$, and $Au_{28}(SCH_3)_{32}$.

| | Structural Region | **$Au_{28}(SCH_3)_{20}$** | **$Au_{36}(SCH_3)_{24}$** | **$Au_{44}(SCH_3)_{28}$** | **$Au_{52}(SCH_3)_{32}$** |
|---|---|---|---|---|---|
| $S_1$ *vs*. $S_0$ | Core | 0.10032 | 0.13180 | 0.20019 | 0.15854 |
| | Motif | 0.31205 | 0.36895 | 0.26944 | 0.23523 |
| | $CH_3$ | 0.38866 | 0.39688 | 0.30688 | 0.26440 |
| | Total | 0.34930 | 0.36714 | 0.28593 | 0.24512 |
| $T_1$ *vs*. $S_0$ | Core | 0.08807 | 0.12063 | 0.18515 | 0.10691 |
| | Motif | 0.28781 | 0.42575 | 0.24153 | 0.17359 |
| | $CH_3$ | 0.32167 | 0.47752 | 0.26146 | 0.19132 |
| | Total | 0.29726 | 0.43572 | 0.24718 | 0.17776 |

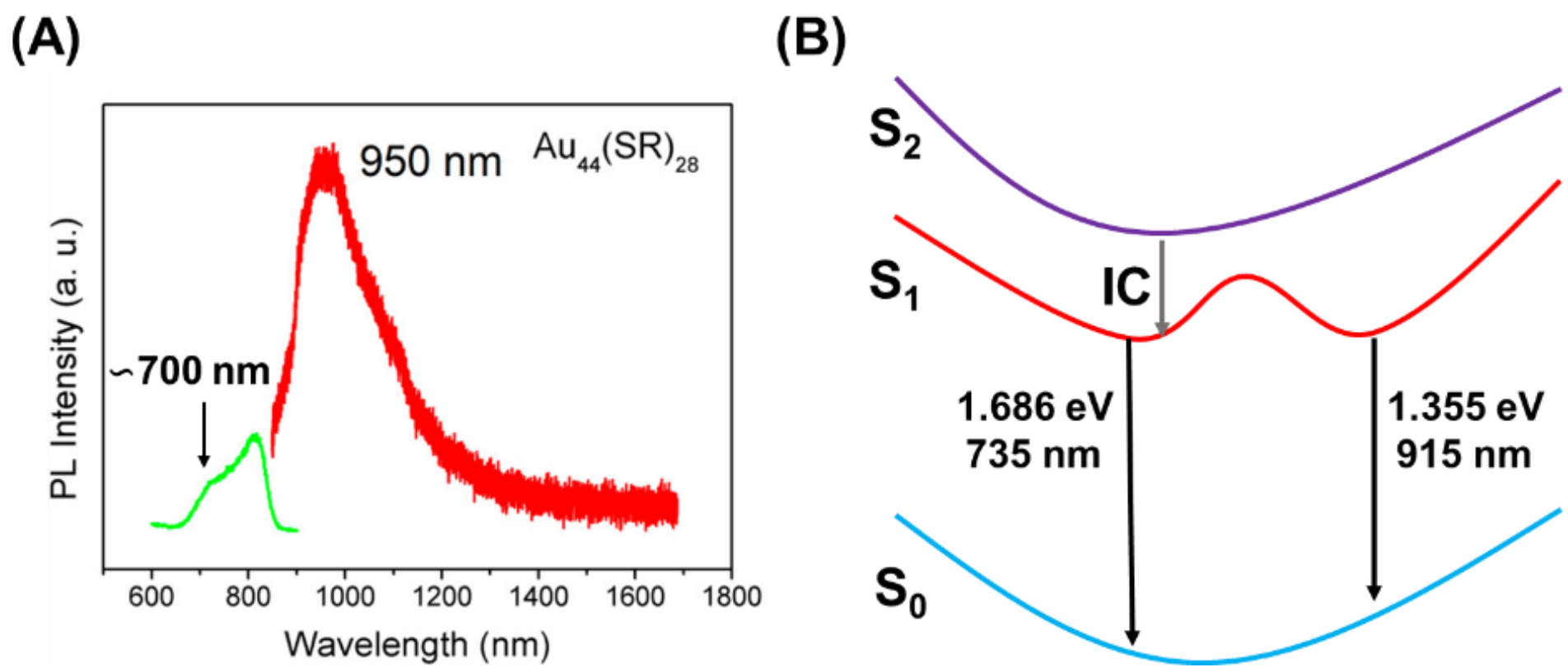


**Figure S1.** **(A)** Photoluminescence (PL) spectra of $Au_{44}(TBBT)_{28}$ (the green and red spectra were collected by two separate detectors: a visible-range detector (up to ~830 nm) and a regular near-infrared one (~900-1700 nm)); **(B)** Schematic illustration of the emission pathway.

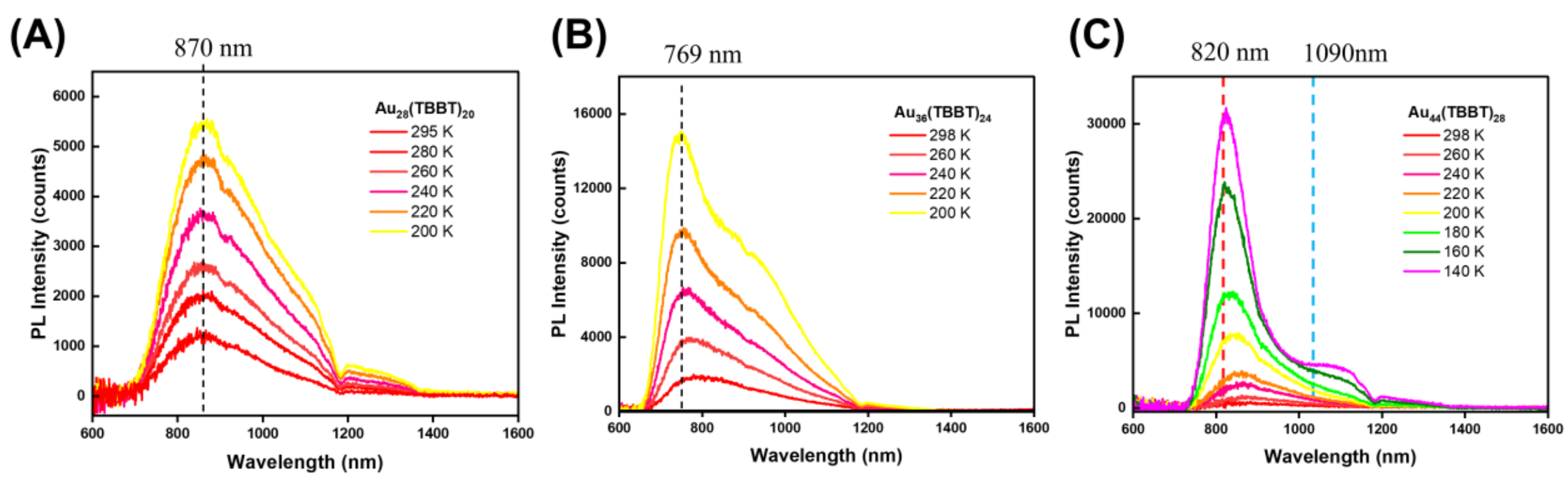


**Figure S2.** Temperature-dependent PL spectra of **(A)** $Au_{28}(TBBT)_{20}$, **(B)** $Au_{36}(TBBT)_{24}$ and **(C)** $Au_{44}(TBBT)_{28}$. The NCs were dissolved in 2-methyltetrahydrofuran (cryogenic solvent, which forms clear glass at cryogenic temperatures). All the PL spectra were collected by a single broadband detector that covers 600-1600 nm, which was cooled to -80 ºC by liquid nitrogen; the NC samples were cooled to various low temperatures by liquid nitrogen as shown in the panels. The single detector (broadband) in these measurements eliminated the balancing and other issues from two separate detectors, hence, the multi-emission nature underlying the PL profile is clearly shown at low temperatures.

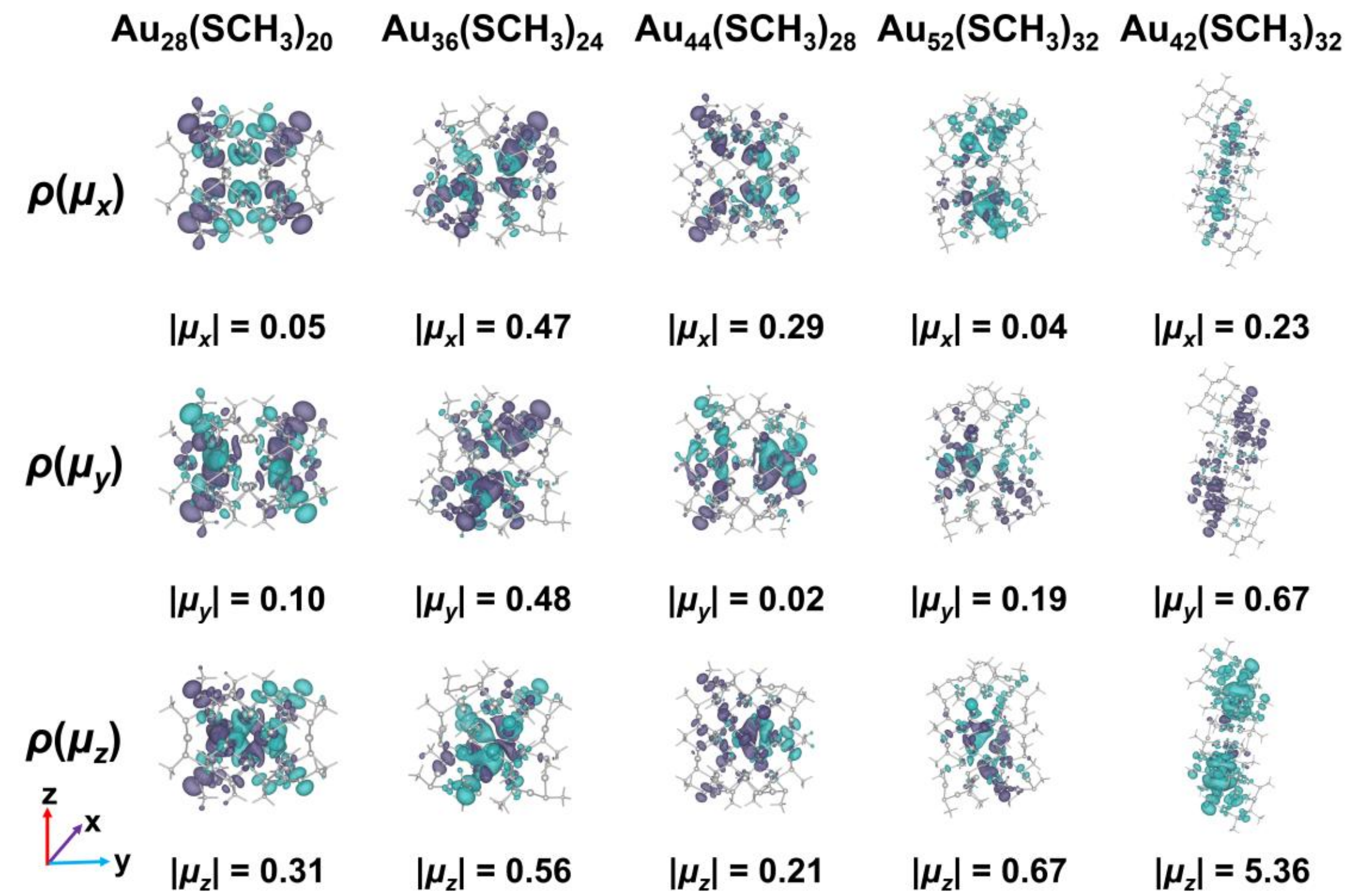


**Figure S3.** Isosurface representations of the transition dipole moment density (TDMD) for $Au_{8n+4}(SCH_3)_{4n+8}$ and $Au_{42}(SCH_3)_{32}$ clusters. Cyan and purple isosurfaces denote positive and negative values of the transition dipole moment density. All isosurfaces are plotted at an identical isovalue of ±0.001 a.u. The clusters framework is shown as light-gray sticks for clarity.

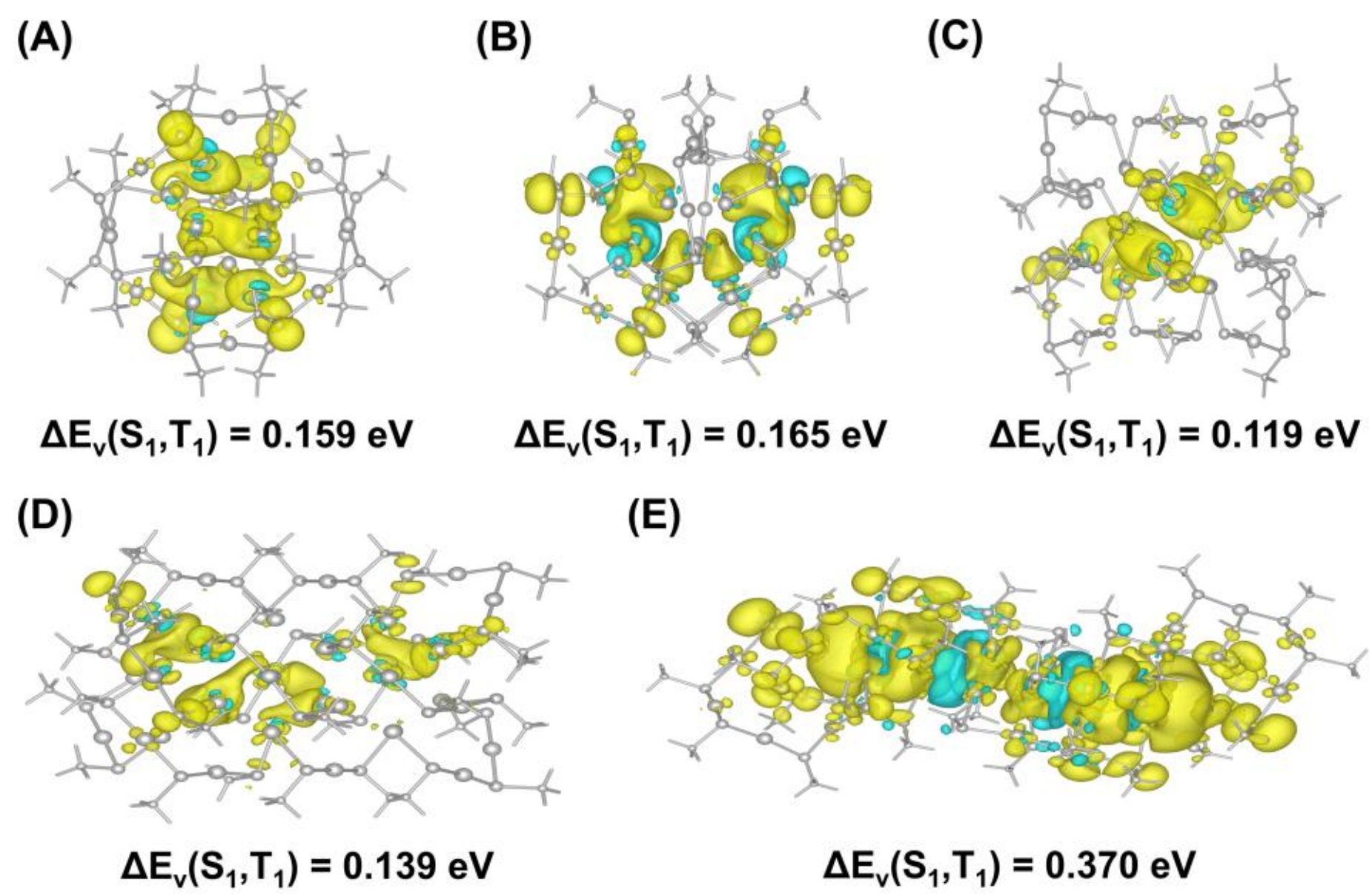


**Figure S4.** Isosurfaces of the exchange energy density for **(A)** $Au_{28}(SCH_3)_{20}$, **(B)** $Au_{36}(SCH_3)_{24}$, **(C)** $Au_{44}(SCH_3)_{28}$, **(D)** $Au_{52}(SCH_3)_{32}$, and **(E)** $Au_{42}(SCH_3)_{32}$. Yellow and cyan denote positive and negative exchange energy density, respectively. All panels are plotted at the same isovalue.

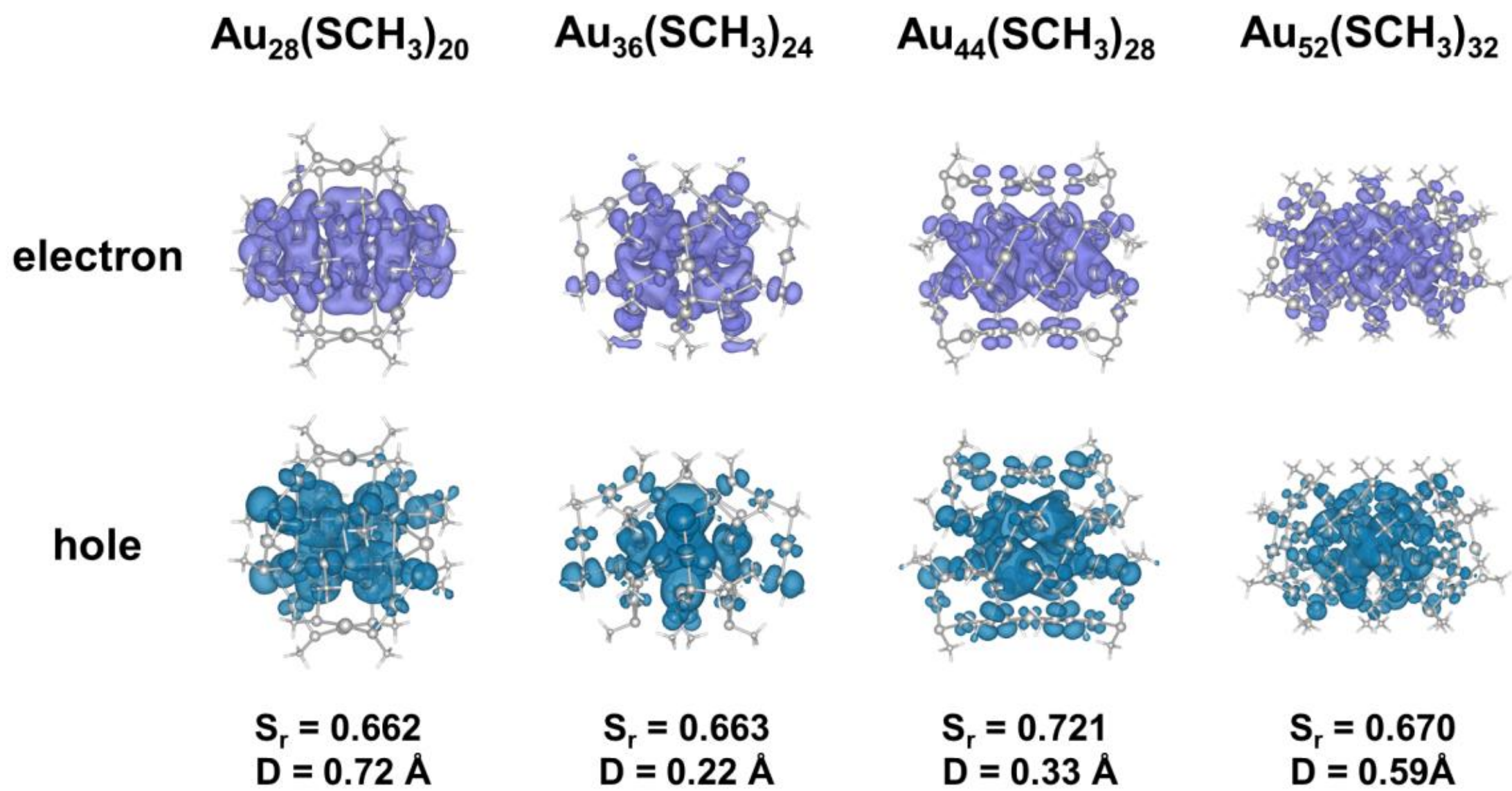


**Figure S5.** Distribution of hole and electron pairs during the $S_0 \rightarrow S_1$ transition at the optimized $S_0$ geometry of $Au_{8n+4}(SCH_3)_{4n+8}$.

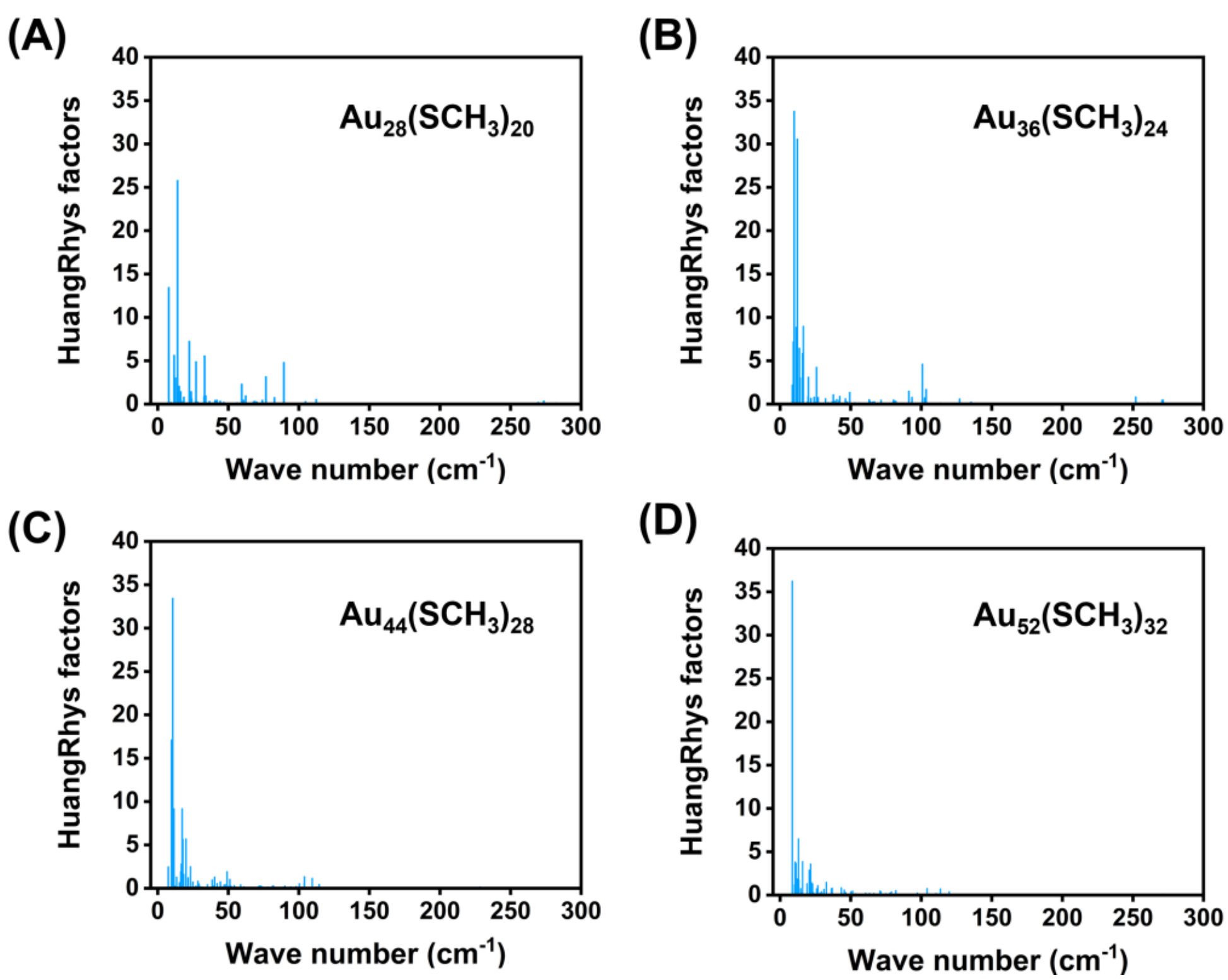


**Figure S6.** Huang–Rhys factors of each normal mode for $Au_{8n+4}(SCH_3)_{4n+8}$.

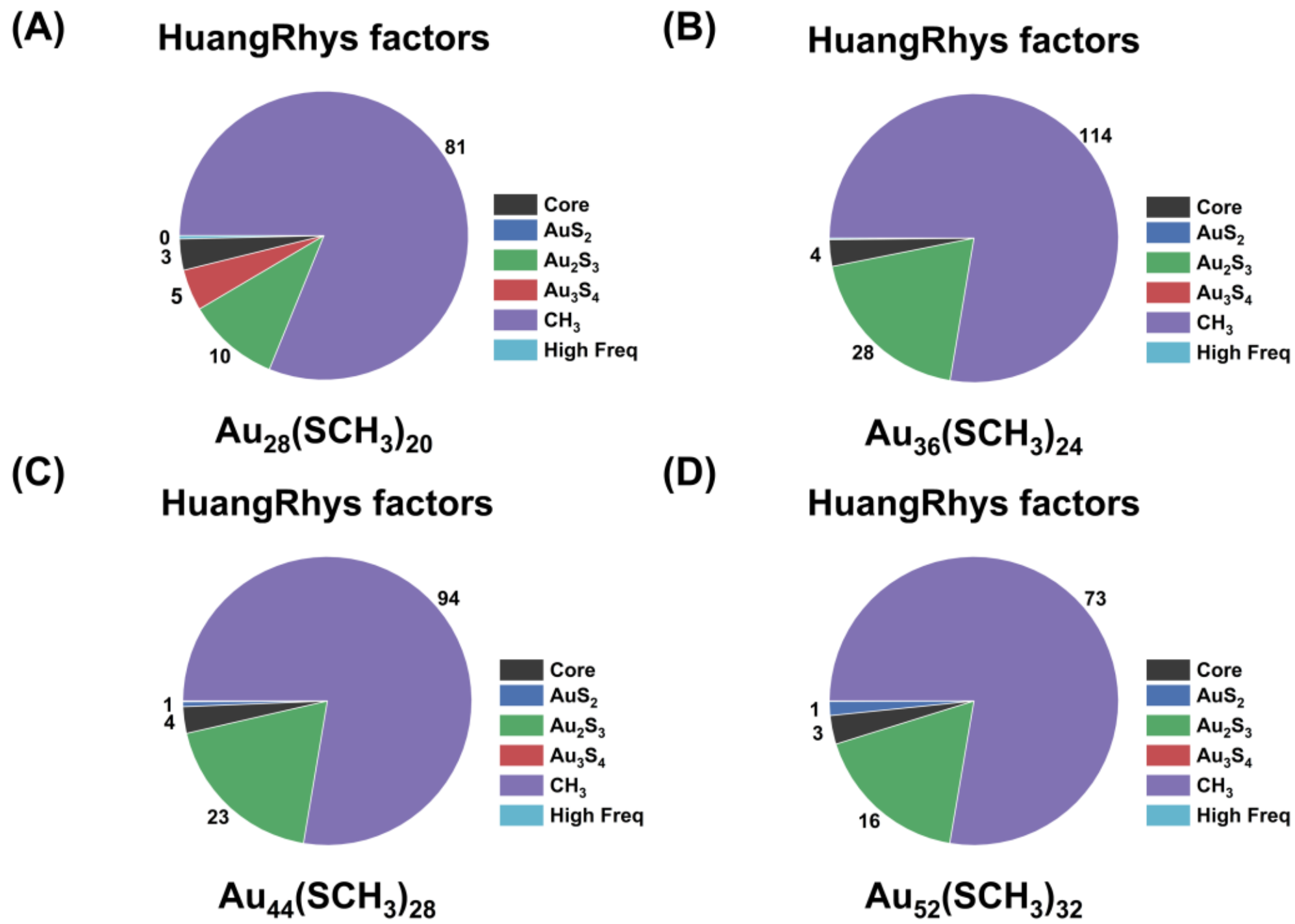


**Figure S7.** Decomposition of the Huang–Rhys factors into contributions from different vibrational mode groups for $Au_{8n+4}(SCH_3)_{4n+8}$. The vibrational modes are classified into core modes, R (i.e., $CH_3$) ligand modes, motif staples modes, and high-frequency modes. The numbers shown on each sector indicate the summed Huang–Rhys factors for the corresponding mode group.

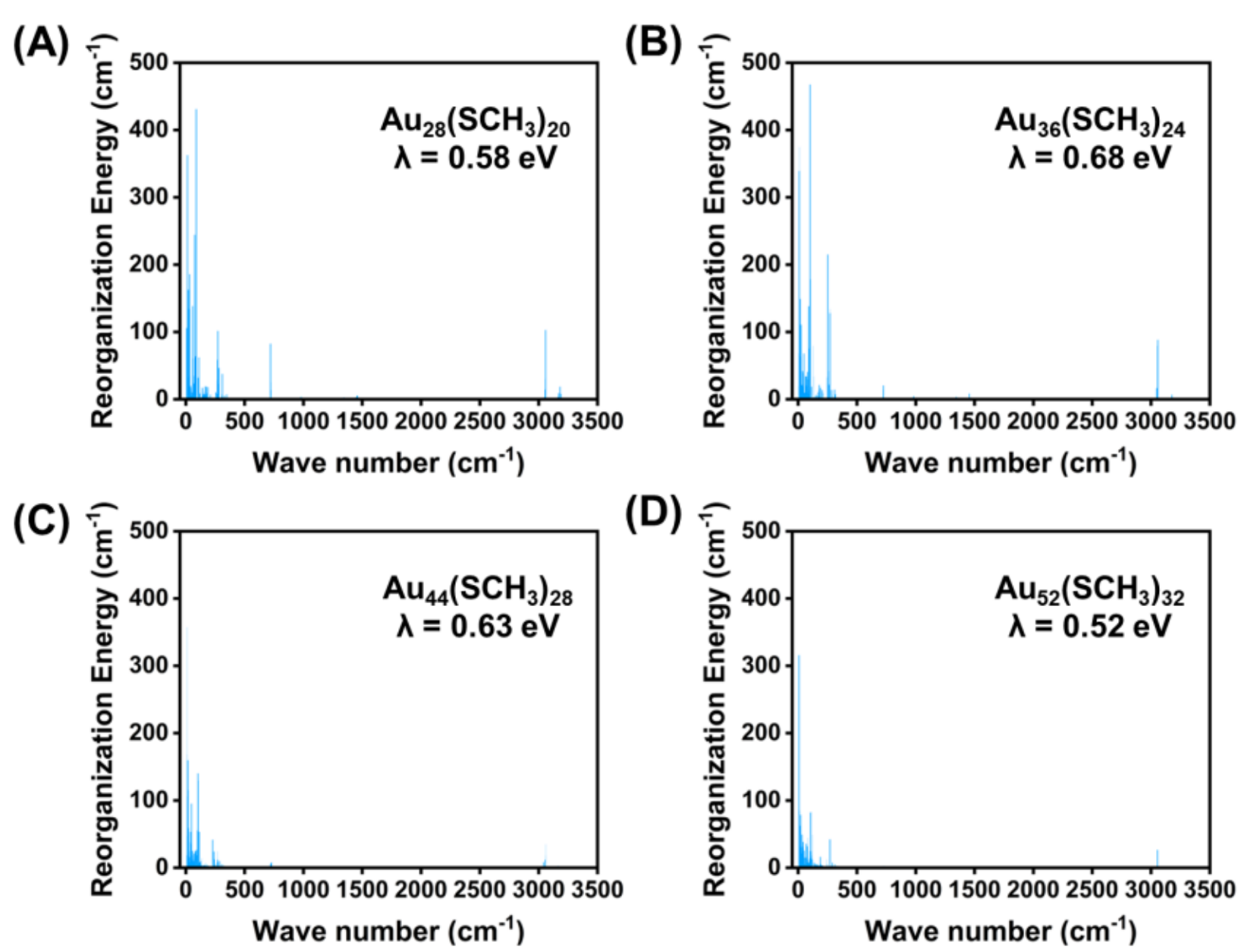


**Figure S8.** Reorganization energies of each normal mode for $Au_{8n+4}(SCH_3)_{4n+8}$.
